# A novel ground truth multispectral image dataset with weight, anthocyanins, and Brix index measures of grape berries tested for its utility in machine learning pipelines

**DOI:** 10.1093/gigascience/giac052

**Published:** 2022-06-14

**Authors:** Pedro J Navarro, Leanne Miller, María Victoria Díaz-Galián, Alberto Gila-Navarro, Diego J Aguila, Marcos Egea-Cortines

**Affiliations:** Escuela Técnica Superior de Ingeniería de Telecomunicación (DSIE), Campus Muralla del Mar, s/n, Universidad Politécnica de Cartagena, 30202 Cartagena, Spain; Escuela Técnica Superior de Ingeniería de Telecomunicación (DSIE), Campus Muralla del Mar, s/n, Universidad Politécnica de Cartagena, 30202 Cartagena, Spain; Genética Molecular, Instituto de Biotecnología Vegetal, Edificio I+D+I, Plaza del Hospital s/n, Universidad Politécnica de Cartagena, 30202 Cartagena, Spain; Genética Molecular, Instituto de Biotecnología Vegetal, Edificio I+D+I, Plaza del Hospital s/n, Universidad Politécnica de Cartagena, 30202 Cartagena, Spain; Sociedad Cooperativa Las Cabezuelas, 30840 Alhama de Murcia, Spain; Genética Molecular, Instituto de Biotecnología Vegetal, Edificio I+D+I, Plaza del Hospital s/n, Universidad Politécnica de Cartagena, 30202 Cartagena, Spain

**Keywords:** Ground truth, grape, computer vision, multispectral, machine learning

## Abstract

**Background:**

The combination of computer vision devices such as multispectral cameras coupled with artificial intelligence has provided a major leap forward in image-based analysis of biological processes. Supervised artificial intelligence algorithms require large ground truth image datasets for model training, which allows to validate or refute research hypotheses and to carry out comparisons between models. However, public datasets of images are scarce and ground truth images are surprisingly few considering the numbers required for training algorithms.

**Results:**

We created a dataset of 1,283 multidimensional arrays, using berries from five different grape varieties. Each array has 37 images of wavelengths between 488.38 and 952.76 nm obtained from single berries. Coupled to each multispectral image, we added a dataset with measurements including, weight, anthocyanin content, and Brix index for each independent grape. Thus, the images have paired measures, creating a ground truth dataset. We tested the dataset with 2 neural network algorithms: multilayer perceptron (MLP) and 3-dimensional convolutional neural network (3D-CNN). A perfect (100% accuracy) classification model was fit with either the MLP or 3D-CNN algorithms.

**Conclusions:**

This is the first public dataset of grape ground truth multispectral images. Associated with each multispectral image, there are measures of the weight, anthocyanins, and Brix index. The dataset should be useful to develop deep learning algorithms for classification, dimensionality reduction, regression, and prediction analysis.

## Context

Traditionally, hyper- or multispectral images (MSIs) have been acquired from satellites or aircraft for the tasks of classification and detection of ground elements [1], vegetation quantification and evolution [2], measurement of ice at the poles [3], or the detection and monitoring of humanmade discharges [4]. The evolution of hyperspectral capture devices based on the decomposition of light in systems with filters on the imaging sensors has introduced notable improvements in spectral sensing. These include a drastically reduced size of the device. The complex calibration process associated with image capturing using linear devices has been eliminated. The number of images per second has been increased. Finally, it is possible to capture up to 25 bands in different spectral ranges in a single shot. These new features allow spectral imaging to expand to new areas of use that were unthinkable a few years ago, such as disease [5] or water stress detection [6] in crops from on-board drones or autonomous robots, food inspection [7], material classification [8], cancer diagnosis [9], or plant phenotyping [10], among others. A difference between hyperspectral and multispectral sensing technology is the extent of the reflectance spectrum captured. In hyperspectral sensing, a contiguous and continuous spectrum is acquired, while in multispectral sensing, only specifically targeted reflectance wavelengths are. In this work, we use the latter technology.

Advances in techniques based on deep learning (DL), inherited from artificial neural networks that mimic the neural behavior of the brain, have managed to outperform humans in automatic pattern recognition systems [11]. Among the different DL techniques, special mention should be given to the convolutional neural networks (CNNs) for their flexibility and scalability when solving problems in the field of computer vision. These networks have obtained excellent results in the detection, classification, and segmentation of images. Furthermore, CNNs can be used to solve regression problems simply by modifying the activation functions of the last layers [12].

Supervised learning algorithms must be trained with ground truth images, that is, images that have been associated with a qualitative or quantitative measurement, also called labels. Obtaining predictive models using multi- or hyperspectral images usually involves 2 stages. First, the images are converted to feature vectors. This process typically involves the use of image semantic segmentation and feature selection or extraction algorithms. The image segmentation can be carried out by filtering the pixels with a reflectance threshold value for one of the channels. This produces a binary image where object and background pixels are identified [13–15]. The process can be done manually using image analysis software such as ENVI [16]. Some feature selection algorithms that are used include competitive adaptive reweighted sampling [13, 15, 17, 18], the successive projection algorithm [13, 15, 17], the genetic algorithm [17], or random frog [14]. The well-known principal component analysis (PCA) and deep neural networks like autoencoders and CNNs can also be used for feature extraction [13]. It is also possible to simply calculate the mean reflectance of object pixels for every channel in the image and use it as features.

Supervised learning algorithms are then used to fit predictive models, such as partial least squares regression, least squares–support vector machines (LS-SVMs), or multilayer perceptron (MLP). Some application examples of these algorithms include the prediction of anthocyanin levels in goji berries (*Lycium ruthenicum*) [13], the pectin content of mulberry [19], the sugar content of wine grape berries [20] and Dangshan pears (*Pyrus* sp.) [17], the pigment levels of spinach leaves [14], the soluble solids content of apple peels [18], and the water and capsaicinoid content of chili peppers [15].

Examples of classification problems related to fruits solved with machine learning algorithms and MSI as input data include the evaluation of injuries in mangoes, with LS-SVM combined with PCA extracted features [21]; the discrimination between naturally and artificially ripened bananas using SVM and the probabilistic collaborative representation classifier [22]; the detection and classification of citrus green mold using linear discriminant analysis [23]; or the discrimination of olive fruits based on their firmness with an MLP [24].

As mentioned above, some algorithms can be used to fit both classification and regression models, such as SVM or MLP. The CNN family of algorithms deserves special attention because they are capable of simultaneously extracting features and fitting a classification or regression model. Thus, when such algorithms are used, there is no need to segment or manually convert the input MSI to feature vectors.

Despite the current importance of image analysis, there are very few ground truth datasets publicly available containing multispectral images. A well-documented dataset corresponding to *Arabidopsis*rosette [25] is found in one source [26]. However, ground truth datasets where the concentration of a chemical or metabolite is coupled to images are nonaccessible, to the best of our knowledge. The multispectral dataset presented in this article is the first that includes multispectral images in the visible and infrared spectrum combined with weight, anthocyanins, and Brix index measurements. The dataset has been designed to be easily used in multispectral image classification with DL methods, dimensionality reduction algorithms based on multispectral images.

## Methods

### Dataset creation

We collected 150 bunches of 5 seedless table grape varieties, *AutumRoyal, Crimson, Itum4, Itum5*, and *Itum9*, to create the dataset. All grapes were collected from the same vineyard, located in the municipality of Alhama de Murcia, in the province of Murcia, in southeast Spain. Grapes were harvested when fully ripe for marketing and export, and samples from the field were used for the study. This was roughly 3 to 4 weeks after veraison.

**Figure 1:**
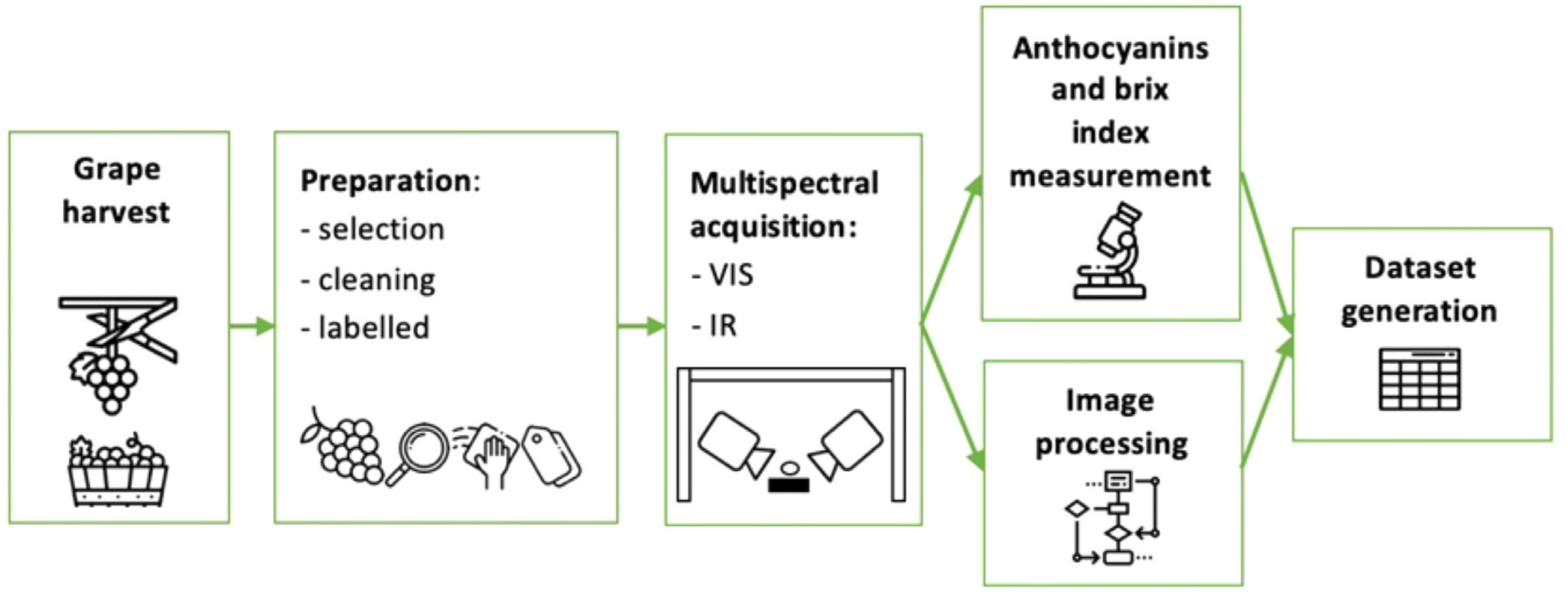
Schematic workflow of the creation of the dataset. Grape bunches from all varieties were harvested after veraison at different months and sent to the laboratory. Individual grapes were selected, cleaned, and labeled prior to data acquisition. Then, the MSIs were captured in the chamber. Afterward, the raw reflectance data were transformed in ready-to-use 3-dimensional arrays, and in parallel, we measured the anthocyanin content and Brix index.

Samples were taken from each bunch in 3 characteristic areas of the bunch, categorized as A—top, B—middle, and C—bottom. The grape berries of every class follow a uniform distribution regarding the area of the bunches they were taken from. Different bunches were used during the sampling to account for the possibility of interbunch variance. We took a total of 1,283 samples: 199 of *AutumRoyal*, 401 of *Crimson*, 84 of *Itum4*, 504 of *Itum5*, and 95 of *Itum9*. Grape berries were cleaned, measured, weighed, and labeled before being introduced into the multispectral chamber to obtain the images. Finally, the labeled berries were sent to the laboratory for anthocyanins and Brix index measurements. The workflow for the creation of the dataset is shown in Fig. 1.

### Multispectral image acquisition

**Figure 2:**
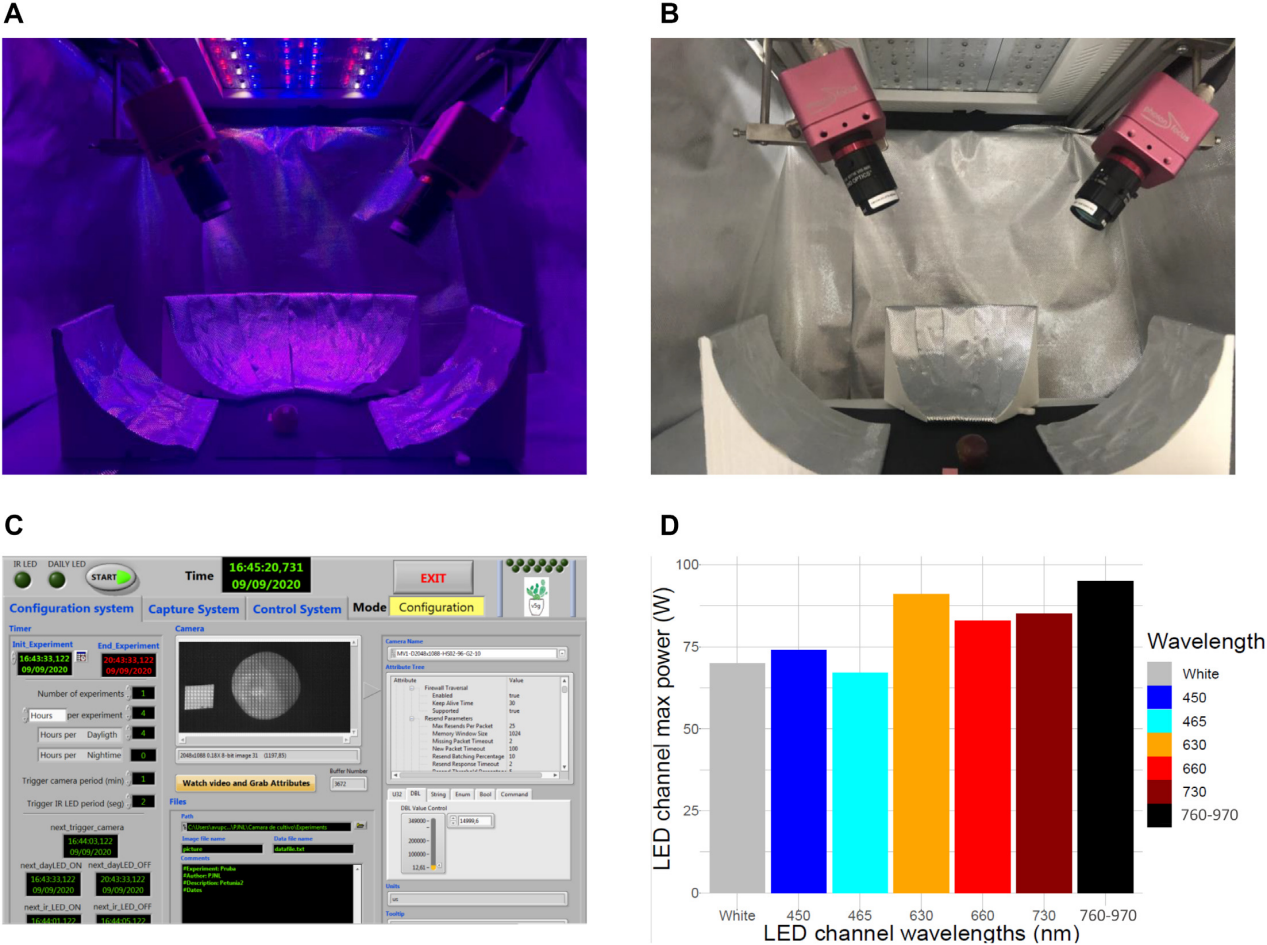
Multispectral chamber: (A) illumination system composed of LEDs of different wavelength; (B) multispectral cameras for visible and infrared acquisition; (C) configuration system panel of software application for the camera setting and to define the parameters of the experiment; and (D) LED illumination system channels’ wavelengths and maximum power in Watts. The 760 to 970 channel is the near infrared channel and comprises LEDs of 760, 800, 820, 840, 880, 910, 940, and 970 nm.

The multispectral acquisition process was carried out in a multispectral chamber specifically developed for this purpose, which is illustrated in Fig. 2. The chamber is composed of:

1. A configurable aluminum structure of size 1,000 × 1,000 × 500 mm^3^. The design of the structure allows easy positioning and placement of different elements, such as reflective panels, cameras, and the illumination system, to prepare different types of experiments.
2. A multispectral illumination system. The illumination has been designed with a cluster of LEDs to cover a broad spectrum of wavelengths from 450 to 970 nm (see Fig. 2D). The power supply to the illumination system is controlled by an analogue electronic controller using a software application.
3. A multispectral acquisition system. The multispectral acquisition system consists of two different snapshot mosaic multispectral cameras from the manufacturer

Photonfocus (Switzerland). The first one (model: MV1-D2048 × 1088-HS03-96-G2) was used for the acquisition of 12 bands in the visible range of 488 to 625 nm, and the second camera (model: MV1-D2048 × 1088-HS02-96-G2) performed the acquisition of 25 bands in the red-infrared range of 676 to 952 nm (see Fig. 2B).

4. A software application system. This has been developed using the LabVIEW programming language. It controls the power supplied to the illumination and multispectral acquisition systems (see Fig. 2C and D).

**Figure 3:**
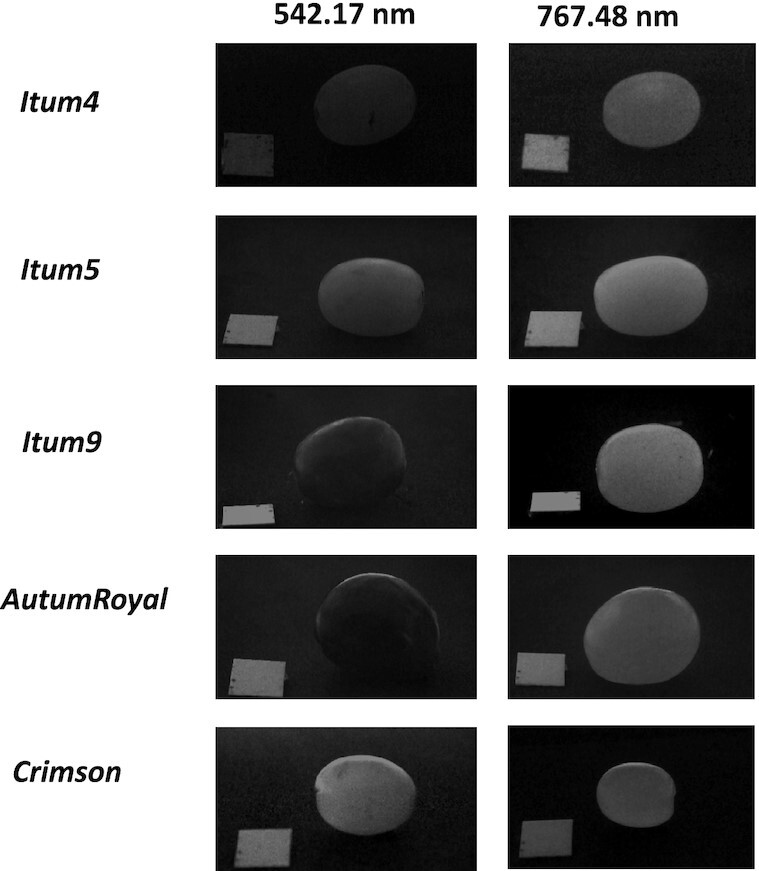
Sample of calibrated images in visible and infrared spectrum. The left column contains the reflectance arrays at 542.17 nm and the right column at 767.48 nm. One random instance of each grape class is shown.

The combined multispectral system acquires 37 raw images (1 byte per pixel) for each grape sample in the spectral range of 430 to 953 nm. We used all the channels, despite increased noise in the reflectance of the 2 end wavelengths, to gather the largest amount of information. The grape samples were captured next to a reference mark with a size of 1 cm^2^ for easy transformation of pixel values to real measurements (see Fig. 3). As images were not taken from a zenith perspective, the square appeared as a rhomboid in the images, thus aiding in the automatic measurement. The multispectral raw image (*I_r_*) is calibrated using 2 reference images captured in two different exposure times (*t*_1,2_) according to equation 1. The dark reference image (*I_d_*) is obtained using a black surface placed at the position of the object to be acquired. The white reference image (*I_w_*) is obtained using a white surface placed at the same position and with the same configuration of the illumination system used for the acquisition.

(1) \begin{eqnarray*}
{I_c} = \frac{{{I_r}({t_1}) - {I_d}({t_1})}}{{{I_w}({t_2}) - {I_d}({t_2})}}
\end{eqnarray*}

The exposure times (*t*_1_, *t*_2_) were selected to avoid saturated pixels in the calibrated image (*I*_c_). Particularly for this dataset, the exposures time was set empirically as *t*_1_ = 15 ms and *t*_2_ = 10 ms.

### Image processing

An example of how the raw MSIs look like is presented in Fig. 3. The noticeable lack of uniformity in the intensity of the images due to the reflectance mainly of the black and red berries was found to be problematic. To segment the grapes and remove unwanted objects from the images, an algorithm based on computer vision techniques has been developed to automatically obtain the segmented image of each grape.

The algorithm consists of the following steps:

1. Extraction of binary patterns from the different types of grapes. This was done selecting 100 images of each variety easy to segment and with a high contrast.
2. Scaling of the binary pattern. From each binary pattern, a set of variations scaling between 0.2 and 20 with step 1.0 is generated, and this is applied to the edge image with the Canny function: {EP_0_,…,EP_*t*_}.
3. MSI preprocessing. To obtain a uniform edge image close to the scaled pattern {EP_0_,…,EP*_t_*}, the following image-processing pipeline was applied over every channel of each MSI:

- Look-at-table with alpha = 2.5
- Gaussian Filter with window 11 × 11
- Adaptative thresholding based on mean with: Size × blockSize = (17.2)
- Morphological CLOSE function with window 3 × 3
- Erase areas less than 500 pixels to avoid small objects
- Canny function edge detector

The result is a set of smooth edges images {SE_0_,…,SE_*n*_} with occlusions per each multispectral image.

4. Matching. A matching function is used to find the grape in the MSI. This is done using multiple instances of the matching function (cv2.matchTemplate(SE_i_, EP_j_, cv2.TM_CCOEFF)) that are invoked between the set of binary patterns {EP_0_,…,EP*_t_*} and the smooth edge images set per each multispectral {SE_0_,…,SE*_n_*}. The matching function supplies a coefficient (TM_CCOEFF) for the correlation between each pair of images {SE_*i*_, EP_*j*_}.
5. Region of interest. The maximum value of the TM_CCOEFF will determine the area where the grape is found. The region of interest, [*y*:*y*+*h,x*:*x*+*w*], will be cropped of the all bands of a specific multispectral image, where (*y, x*) is the upper left corner of the rectangle with greater TM_CCOEFF, *h* is the pattern height, and *w* is the pattern width.

**Figure 4:**
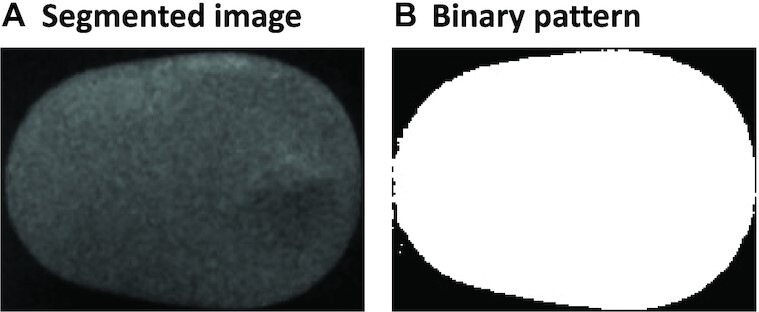
Result of the multispectral image segmentation algorithm for a *Crimson* grape. (A) Final segmented image. (B) Binary pattern extracted.

An example of a segmented multispectral image obtained with this algorithm is shown in Fig. 4 (only one reflectance channel shown).

6. Save segmented images and go to step 1.

This algorithm had to be developed because a simple threshold segmentation with the raw images as input would not work with the darker berries, namely, those of *AutumRoyal* class.

### Anthocyanins and Brix index measures

Anthocyanins were quantified as described previously [27]. Total anthocyanins were extracted with 5 ml of an acidic methanol (0.1%) solution, CH_3_OH:HCl:H_2_O (70:0.1:29.9 v/v/v). Samples were incubated at 4°C in darkness for 24 hours. Then, anthocyanins were measured using 2 different absorbances (530 and 657 nm) with a spectrophotometer (ion UV 1600, US, UV-2401-PC UV-VIS Spectrophotometer, Shimadzu Corporation, Kyoto, Japan). The amount of anthocyanin was obtained by using equation 2:

(2) \begin{eqnarray*}
{Q_{total}}\,\,{\rm anthocyanin} = \left( {{A_{530}} - 0.25 \times {A_{657}}} \right)/FW
\end{eqnarray*}

where A_530_ and A_657_ are the absorbances obtained with the spectrophotometer with a wavelength of 530 nm and 657 nm, respectively. FW is the fresh weight of the sample.

We measured Brix index with a digital refractometer (ATAGO PAL-1,Tokio, Japan) using the grape juice extracted from each berry [28].

## Data Validation and Quality Control

### Dataset structure

The data, which are stored in *gigadb.org*, consists of a total of 1,283 multidimensional arrays in TIF format, compressed in a single zip file. There are 5 different grape varieties in the dataset. The images of *AutumRoyal, Crimson*, and *Itum5* classes have been obtained in several batches on different days. In the case of *Crimson* and *Itum5*, images were acquired in different months. This is important because grape attributes are not uniform all over the year and in fact vary depending on the month. Table 1 shows the composition of the dataset, that is, the number of instances of each grape class as well as over how many days the images were obtained and in which month(s). The *AutumRoyal* is a black grape, *Crimson* is green to lightly red, *Itum4* and *Itum5* are green, and *Itum9* is dark red. The size of this dataset is comparable to the one in Ramos et al. [29], who used 1,260 MSI of grapes of 2 varieties to adjust a classifier capable of predicting the ripeness of grape berries.

**Table 1:** Structure of the dataset

Grape type	Number of images	Acquisition batches	Month(s)
AutumRoyal	199	2	November
Crimson	401	4	September, October, November, December
Itum4	84	1	September
Itum5	504	5	October, November, December
Itum9	95	1	Sept.

For each grape variety present in the dataset, the following information is presented: the number of instances, over how many days they were acquired, and in which month(s).

Each one of the arrays in the dataset corresponds to an MSI, with the following dimensions, in pixels: 140 (height) × 200 (width) × 37 (number of channels/depth). The first 12 channels correspond to reflectance in the visible range of the electromagnetic spectrum and the last 25 ones to near infrared (NIR) reflectance. The specific wavelength of every channel is shown in Table 2.

**Table 2:** Multispectra image wavelengths

Channel	Wavelength (nm)	Channel	Wavelength (nm)	Channel	Wavelength (nm)
1	488.38	14	689.83	27	866.68
2	488.58	15	714.87	28	876.49
3	503.59	16	729.06	29	885.18
4	516.60	17	741.80	30	894.26
5	530.62	18	755.44	31	908.58
6	542.17	19	767.48	32	916.63
7	567.96	20	781.25	33	925.36
8	579.29	21	792.57	34	931.99
9	592.89	22	802.25	35	938.48
10	602.88	23	823.75	36	946.14
11	616.59	24	835.10	37	952.76
12	625.71	25	845.81		
13	676.25	26	856.52		

In addition to the images, a ground truth for specific variables in the format of a tab separated text file (.txt) is stored in the repository. For every grape in the dataset, the value of the following variables is present in this file: the Brix index, weight expressed in grams, the amount of anthocyanins expressed in milligrams per kilo of fresh weight, the type of grape, and finally an identification of the measured batch. As an overview of this file, the first 5 rows are shown in Table 3.

**Table 3:** Overview of the ground truth table file with the first 5 rows presented

ArrayName	Brix.Index	Grams	Anthocyanins.mg.Kg.FW	Type	Measure
Crimson_37bands_1.TIF	21.7	0.028	4.070	Crimson	Crimson September
Itum9_37bands_1.TIF	18.5	0.100	33.239	Itum9	Itum9 September
Itum5_37bands_1.TIF	20.7	0.066	0.370	Itum5	Itum5 October
AutumRoyal_37bands_1.TIF	11.7	0.077	14.892	AutumRoyal	AutumRoyal 1 November
Itum9_37bands_2.TIF	18.7	0.0882	37.757	Itum9	Itum9 September

### Dataset visualization

The first analysis was a dimensionality reduction with the PCA algorithm to gain insight of the internal structure of the dataset. To do that, the dataset was compressed from a collection of multispectral images to a 2-dimensional table. This was done by threshold segmentation of the multispectral images and then calculating the mean reflectance across all object pixels for every channel and image. The resulting table has as many rows as there are images in the dataset and as many columns as reflectance channels. For the image segmentation, the channel 22 (802.25 nm) and the threshold value 25 were used. These parameters were both empirically selected. Once the dataset had been compressed, the PCA algorithm was applied to it.

**Figure 5:**
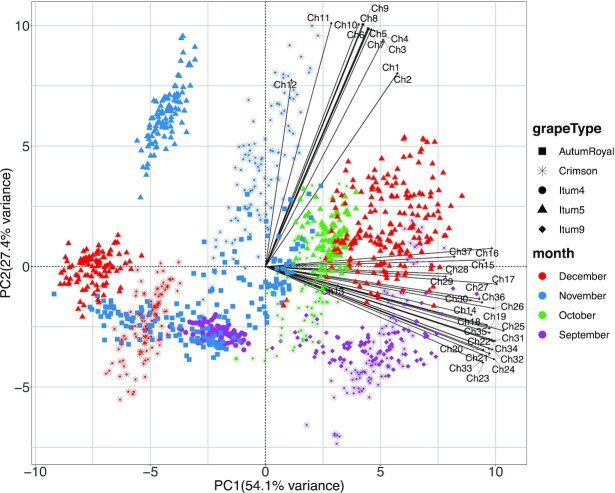
Principal components analysis scatter and variable plot (biplot) of the dataset. The class of the instances is represented by the shape of the points and the month when the image was acquired by the color. The variables are represented as black lines with their names next to them. The reflectance of each channel is presented in Table 2.

The first two principal components (PCs) explain up to 84% of the total variance of the dataset. More specifically, the first PC was mostly a product of the NIR reflectance channels, and the second PC was formed by the visible range channels. The first PC itself accounts for 54% of the total variance and, as shown in Fig. 5, can separate most of the instances. This suggests that most of the reflectance of the grape berries is formed by NIR radiation and thus it is within this range of the spectrum where the different varieties can be best discriminated.

Fig. 5 illustrates substantial intragroup variance for *AutumRoyal, Crimson*, and *Itum5* grape types, while it is less pronounced in *Itum9* and *Itum4*. As mentioned earlier, the most likely explanation for this phenomenon is that the images of some classes were captured over more than 1 day and in different months. This is particularly remarkable for *Itum5* and *Crimson* because they were measured over 3 and 4 months, respectively. By contrast, *Itum9* and *Itum4* were measured in only 1 month, and they cluster much closer together.

**Figure 6:**
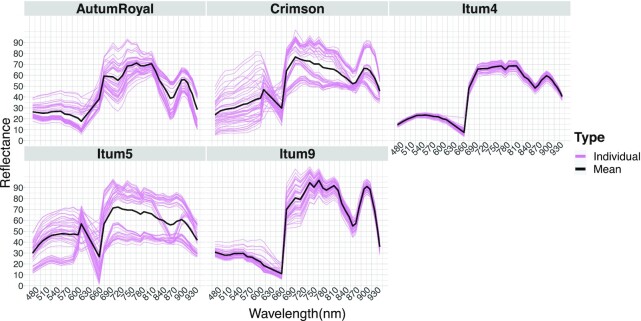
Reflectance spectrograms of every grape class. For each class, the mean spectrogram across all instances is presented as well as the individual spectrogram of 50 randomly selected instances.

We also assessed the differences between every grape class via their reflectance spectrogram comparison (Fig. 6). In accordance with Fig. 5, most of the reflectance was found to be in the NIR range, and this was also the region with the most intergroup variance. We selected grapes randomly using the Python library Numpy random sampling method. By plotting the spectrogram of each class, we could also see the heterogeneity of *AutumRoyal, Crimson* and *Itum5* (Fig. 6).

The spectra obtained from grape berries differ significantly from the better-known leaf spectra. This is due mostly to the differences in chlorophyll content of the tissues. Indeed, the reported concentration of chlorophyll in grape berries at harvest is 1,000-fold lower than leaves of spinach, lettuce, or pakchoi [30, 31].

**Figure 7:**
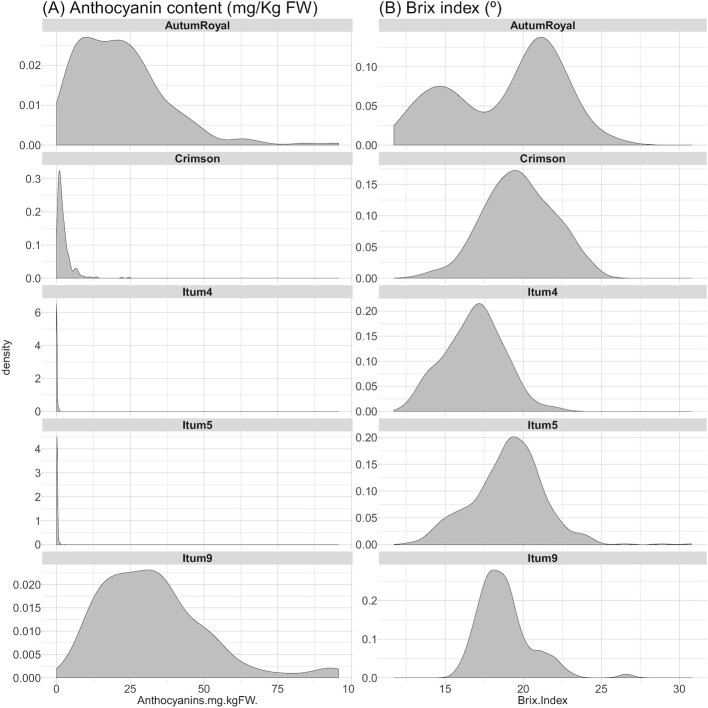
Density plots of the distribution of the variables anthocyanin content (A) and Brix index (B) for each individual class.

Finally, the distribution of the continuous variables present in the ground truth table file was analyzed, namely, anthocyanin content in mg/kg fresh weight and Brix index degrees (Fig. 7). According to the Shapiro–Wilk tests, anthocyanins were not normally distributed in the varieties analyzed. *Itum5, Itum4*, and *Crimson* had leptokurtic distributions while *AutumRoyal* and *Itum9* had platykurtic distributions. The anthocyanin ranges also differed between each grape class. Both *Itum4* and *Itum5* had a very short range, centered on zero [0–0.98] and [0.0073–2.96], respectively, while it was much broader in the cases of *AutumRoyal* [0.63–95.23] and *Itum9* [2.78–95.60]. Finally, *Crimson* ranged between 1.45e-4 and 2.45 mg/kg fresh weight. These differences were due to the different pigmentation levels of the grapes. *Itum4* and *Itum5* are both green grapes and thus lack anthocyanin pigments in the skin of the berries. In contrast, *Crimson* is lightly red colored and therefore possesses some pigmentation, while *AutumRoyal* and *Itum9* are black or dark red and have the highest level of anthocyanins in their skin.

The Brix index distributions were less skewed than anthocyanins (Fig. 7). *Crimson* and *Itum4* were normally distributed, according to the result of Shapiro–Wilk tests. Their ranges were also more similar than was the case with the anthocyanin content: [12.8–22] for *Itum4*, [12.6–30.8] for *Itum5*, [15.9–26.6] for *Itum9*, 25.3-braquet for Crimson. Interestingly, *AutumRoyal* and, to a lesser extent, *Itum9* showed a bimodal distribution. As these grapes were measured over a single month each, this may indicate differing levels of ripening in the trusses analyzed (Fig. 7).

## Potential usage of dataset

### Dataset utility in machine learning pipelines

To assess the usefulness of this ground truth data set in machine learning pipelines, we tried unsupervised and supervised pipelines, fitting classification models. We used a K-means clustering algorithm for the unsupervised pipeline and multilayer perceptrons (MLP) and convolutional neural networks with 3-dimensional kernels (3D-CNN) for the classification models. The goal of the classifiers is to predict the class of the grapes.

Two neural network algorithms were used to fit classification models: MLP and 3D-CNN. MLP requires the data in 2-dimensional table form, the same one that was used as input for the PCA algorithm.

The second one can use the 3-dimensional arrays of the dataset directly as input but requires the usage of data augmentation techniques, such as those provided in the Python 3 library Albumentations [32].

The MLP used consisted of a simple stacking of 4 dense layers with 32, 24, 16, and 8 nodes each, plus a final output layer with 5 nodes. Between each dense layer, a batch normalization layer and the ReLU activation function were inserted. After the output dense layer, we used the softmax activation function. This network is quick to train, but the input dataset must be preprocessed beforehand, via spatial and spectral compression.

The 3D-CNN architecture employs two 3-dimensional convolutional blocks with convolutional layers that use dilated 3-dimensional kernels [33]. It also uses mean and average 3-dimensional pooling layers between these blocks and a total of 2 dense layers. This network not only requires a great number of images to be trained (hence the need of data augmentation techniques) but is also slow. It needs to store in memory tensors of considerable size, which is computationally heavy. The network architecture is summarized in Fig. 9.

To train both networks, the data were split between 3 separate subsets called train, validation, and test, each one having respectively the 50%, 25%, and 25% of the total instances. The split was not done randomly; instead, the class distribution was preserved in all subsets, so that in each one, there was the same proportion of grapes. The train subset is used to fit the model, the validation is used for testing it while it is being fit, and the test subset is used after the fit has been completed to get a final performance value. By splitting the data like this, the leakage of information between subsets is avoided. In the case of the model fitted with the 3D-CNN algorithm, data augmentation was applied to both train and validation subsets but not to the test subset. The data augmentation consisted of affine transformations, vertical and horizontal flippings, and lightly altering the pixel values via contrast and brightness changes. The transformations applied were carefully selected to avoid distorting the reflectance information contained in the images. The flipping of images and affine transformation do not change the pixel reflectance values contained in the pixels. The contrast and brightness do change the pixel values, but its range was limited to minimize any possible hindrance in the learning process of the classifiers. The precise values of the transformation ranges are identical to those described in the literature [33].

In both cases, a perfect (100%) classification accuracy was reached, which validates the quality of this dataset to fit classification models with complex and simple network architectures. The success of these algorithms is likely due to the fact that they are able to find and exploit nonlinear relationships between the independent variables (i.e., the reflectance spectra).

Indeed, we have also fit a classifier using a simpler algorithm, namely, SVM with linear kernel, and we obtained a modest 0.679 accuracy as the best result. This indicates the need for higher complexity learning algorithms to fit models capable of generalizing with these data.

The visible and infrared arrays of the MSI were not identical with regard to the spatial positions of the object pixels, as they were captured with 2 separate cameras. This was not an obstacle for the fitting of classification models.

**Figure 8:**
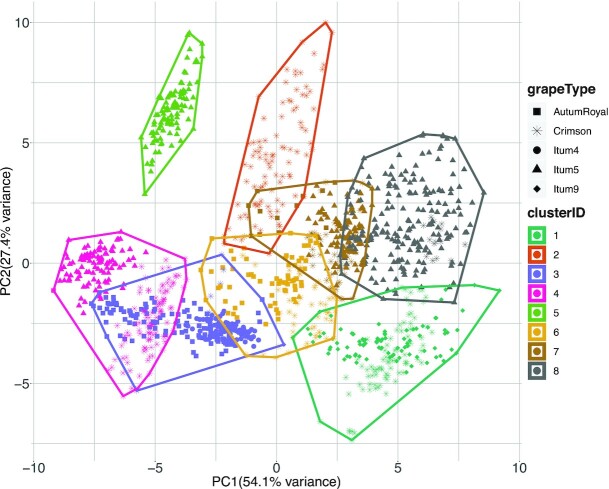
PCA scatterplot with color coding for the clusters calculated with the K-means algorithm. The shape of the points represents the grape class.

The result of the K-means clustering (with K = 8 empirically selected) showed that grapes were not cleanly separated. Interestingly, the month in which the images were acquired does indeed introduce significant differences between grapes of the same type. This was evidenced by the fact that the instances of most classes were distributed across different clusters and that several clusters contain instances of different classes. In particular, *AutumRoyal* was split into 2 different clusters. *Crimson* was distributed across mainly 4 clusters, but only one of these clusters was composed entirely of *Crimson* instances. *Itum4* instances were all located in one cluster, together with *AutumRoyal* instances. *Itum5*, like *Crimson* was split across 4 clusters, but only one of them was composed entirely of instances of this class. Finally, for *Itum9*, only 1 cluster contained all the instances, together with *Itum5*. Except for *AutumRoyal*, the number of clusters containing all instances of a given grape class coincided with the number of months over which the images of that class were acquired. The results were visualized over the same PCA scatterplot already shown in Fig. 5 and presented in Fig. 8. These clustering results indicate that a supervised learning algorithm may find problems fitting a classification algorithm, because instances labeled equally can have quite different reflectance spectra depending on the month when the image was captured. In contrast, neural networks achieve 100% accuracy.

**Figure 9:**
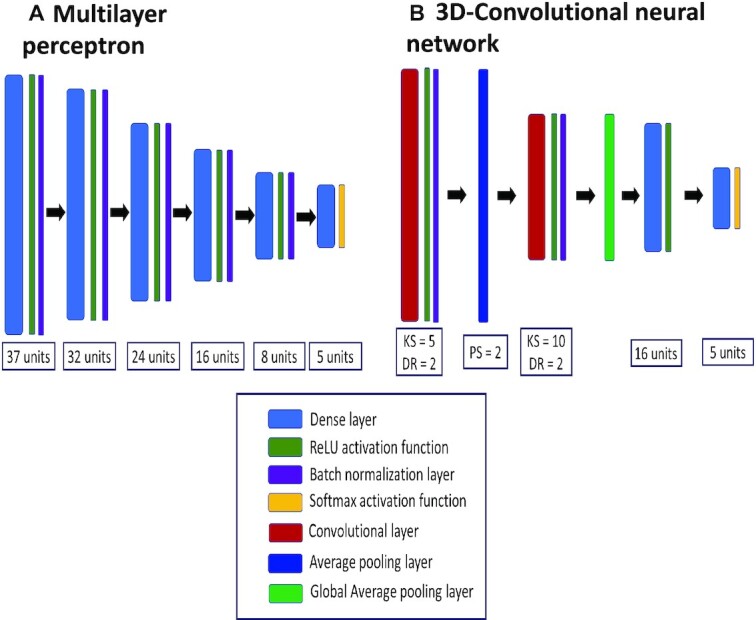
Schematic representation of the neural networks employed to fit classification models. The term unit that appears in the multilayer perceptron (A) and 3-dimensional convolutional neural network (B) refers to the number of neurons of a dense layer. In (B), the following abbreviations are used: KS equals kernel size of a convolutional layer; DR equals dilation rate of the kernels of a convolutional layer, and PS equals pool size of a pooling layer.

An unexpected result of our analysis was the capacity to identify differences between berries based on time of harvest or position in the truss. This explains the significant intragroup variance in some grape classes. Those were not an obstacle for the neural networks used as classification algorithms. Furthermore, our dataset reflects the reality encountered when using agricultural products (i.e., a large variance due to a combination of environmental, ontogenic, and genetic factors).

In addition to classification and clustering, we have also tried to fit regression models capable of predicting either the anthocyanin content or the Brix index. However, we were unsuccessful in our attempts. We believe that the structure of the data prevents the algorithms to extract meaningful relations between the reflectance and the values of the continuous variables presented (anthocyanin content and Brix index). The distributions of anthocyanin content are too different between grape classes. More than three-quarters of all grapes measured had little to undetectable anthocyanin levels (*Itum5, Itum4*, and *Crimson*), while the remaining classes had very high levels (*AutumRoyal* and *Itum9*). Hence, the algorithms were challenged to fit a model capable of generalizing. Restricting the problem to only one or a few classes was of no use because the number of instances turned out to be too low for the learning algorithms.

The Brix index posed a different problem to fit regression models. In this case, the distribution of this variable is very similar for every grape class of the dataset. This causes the algorithms to fit a model that systematically predicts the global mean of this variable. They are not capable of linking the information contained in the spectra to the Brix index.

We have tried 2 additional algorithms, namely, partial least squares regression and SVM, alongside the neural networks presented in the study adapted for regression problems, and none of them were able to successfully fit a regression model. The highest determination coefficient (*R*^2^) was 0.53 for anthocyanin and 0.24 for the Brix index (data not shown).

We have obtained 1,238 multispectral images from grape berries, comprising 37 channels, thus creating a multidimensional array of 45,806 images. Coupled to each grape, there are additional data, such as weight, anthocyanin content, and Brix index. To the best of our knowledge, this is the first dataset of ground truth multispectral images of fruits to be made publicly available. We propose it as a benchmark for the plant phenotyping community that uses multispectral images to test different classification algorithms on it.

## Availabiliy of source code and requirements

Project name: 3DeepM

Project home page: https://github.com/AlbertoGilaNavarro/3DeepM [33]

Operating system: Platform independent

Programming languages: Python

Other requirements: Python version 3.6.8 or higher, Tensorflow version 2.4.1 or higher, Numpy version 1.19.5 or higher, Pandas version 1.1.5 or higher, Matplotlib version 3.3.4 or higher, Albumentations version 0.5.2 or higher, ComputerVision2 (cv2) version 4.5.1 or higher, Skimage version 0.17.2 or higher, Imutils version 0.5.4 or higher

License: GNU General Public License version 3

In our home page, the functions for the segmentation process of grape berries and the architectures of the neural networks used in the technical validation, together with the usage example of model training and validation scripts, are publicly available.

## Data availability

All data further supporting this work, including snapshots of our code, are openly available in the GigaScience repository, GigaDB [34].

## Authors' contributions

P.J.N. and M.E.C. designed the experiments, supervised the research, and obtained funding. L.M. and M.V.D.G. obtained data. D.A. grew, obtained biological samples, and selected representative samples. P.J.N. and A.G.N. analyzed data. P.J.N., L.M., A.G.N., and M.E.C. wrote the first draft. All authors corrected and approved manuscript.

## Competing interests

D.A. is a table grape grower. D.A. had no direct influence on the outcome of the experiments.

The rest of the authors declare no conflict of interest.

## Supplementary Material

giac052_GIGA-D-22-00030_Original_Submission

giac052_GIGA-D-22-00030_Revision_1

giac052_GIGA-D-22-00030_Revision_2

giac052_Response_to_Reviewer_Comments_Original_Submission

giac052_Response_to_Reviewer_Comments_Revision_1

giac052_Reviewer_1_Report_Original_Submission Chris Armit -- 3/8/2022 Reviewed

giac052_Reviewer_2_Report_Original_Submission I.G.C.P. Melo-Pinto -- 3/22/2022 Reviewed

giac052_Reviewer_2_Report_Revision_1 I.G.C.P. Melo-Pinto -- 4/13/2022 Reviewed

giac052_Reviewer_3_Report_Original_Submission Stefan Paulus, Ph.D. -- 3/24/2022 Reviewed

giac052_Reviewer_3_Report_Revision_1 Stefan Paulus, Ph.D. -- 4/19/2022 Reviewed
