# Supplementary material for: A novel ground truth multispectral image dataset with weight, anthocyanins, and Brix index measures of grape berries tested for its utility in machine learning pipelines
Source: Gigascience. 2022 Jun 14;11:giac052. doi: 10.1093/gigascience/giac052 (PMC9197681; doi:10.1093/gigascience/giac052)
Supplement: giac052_GIGA-D-22-00030_Revision_2 [file giac052_giga-d-22-00030_revision_2.pdf]

GigaScience

A novel ground truth multispectral image dataset with weight, anthocyanins and brix index measures of grape berries tested for its utility in machine learning pipelines

--Manuscript Draft--

**Manuscript Number:**	GIGA-D-22-00030R2
**Full Title:**	A novel ground truth multispectral image dataset with weight, anthocyanins and brix index measures of grape berries tested for its utility in machine learning pipelines
**Article Type:**	Data Note
**Funding Information:**	Ministerio de Economía, Industria y Competitividad, Gobierno de España (BFU 2017-88300-C2-1-R) — Prof.Dr. Marcos Egea-Cortines<br>Ministerio de Economía, Industria y Competitividad, Gobierno de España (BFU 2017-88300-C2-2-R) — Dr Pedro J. Navarro<br>Centre for Industrial Technological Development (5117/17CTA-P) — Prof.Dr. Marcos Egea-Cortines<br>Fundación Séneca (19895/GERM/15) — Dr Pedro J. Navarro

Abstract:

Background

The combination of computer vision devices such as multispectral cameras coupled with Artificial Intelligence has provided a major leap forward in image-based analysis of biological processes. Supervised Artificial Intelligence algorithms require large ground truth image datasets for model training, which allows to validate or refute research hypotheses and to carry-out comparisons between models. However, public datasets of images are scarce and ground truth images are surprisingly few considering the numbers required for training algorithms.

Results

We created a dataset of 1283 multidimensional arrays, using berries from five different grape varieties. Each array has 37 images of wavelengths between 488.38nm and 952.76nm obtained from single berries. Coupled to each multispectral image we added a dataset with measurements including, weight, anthocyanin content and Brix index for each independent grape. Thus, the images have paired measures creating a ground truth dataset. We tested the dataset with two neural network algorithms: multilayer perceptron (MLP), three-dimensional convolutional neural network (3D-CNN). A perfect (100% accuracy) classification model was fit with either the MLP or 3D-CNN algorithms.

Conclusions

This is the first public dataset of grape ground truth multispectral images. Associated with each multispectral image there are measures of the weight, anthocyanins, and brix index. The dataset should be useful to develop deep learning algorithms for classification, dimensionality reduction, regression, and prediction analysis.

Context

Traditionally, hyper o multispectral images (MSI) have been acquired from satellites or aircraft for the tasks of classification and detection of ground elements [1], vegetation quantification and evolution [2], measurement of ice at the poles [3], or for the detection and monitoring of man-made discharges [4]. The evolution of hyperspectral capture devices based on the decomposition of light in systems with filters on the imaging sensors has introduced notable improvements in multispectral technology. These include a drastically reduced size of the device. The complex calibration process associated with image capturing using linear devices has been eliminated. The number of images per second has been increased. Finally, it is possible to capture up to 25 bands in different spectral ranges in a single shot. These new features allow spectral

Powered by Editorial Manager® and ProduXion Manager® from Aries Systems Corporation

	imaging to expand to new areas of use that were unthinkable a few years ago, such as: disease [5] or water stress detection [6] in crops from on-board drones or autonomous robots, food inspection [7], material classification [8], cancer diagnosis [9], or plant phenotyping [10], among others.
**Corresponding Author:**	Marcos Egea-Cortines, PhD<br>Universidad Politecnica de Cartagena<br>Cartagena, Murcia SPAIN
**Corresponding Author Secondary Information:**	
**Corresponding Author's Institution:**	Universidad Politecnica de Cartagena
**Corresponding Author's Secondary Institution:**	
**First Author:**	Marcos Egea-Cortines, PhD
**First Author Secondary Information:**	
**Order of Authors:**	Marcos Egea-Cortines, PhD
	Pedro J. Navarro, Professor
	Leanne Miller
	María Victoria Díaz-Galián
	Alberto Gila-Navarro
	Diego J. Aguila
**Order of Authors Secondary Information:**	
**Response to Reviewers:**	Thanks for the review. Concerning the veraison exact dates, there is no such a thing in large orchards. Grapes show a veraison time for a given variety that may take up to a month, depending on the terroir. Thus we think that stating that grapes were taken to the lab when ripe is the best approximation to this issue.
**Additional Information:**	
**Question**	**Response**
Are you submitting this manuscript to a special series or article collection?	No
**Experimental design and statistics**<br><br>Full details of the experimental design and statistical methods used should be given in the Methods section, as detailed in our Minimum Standards Reporting Checklist. Information essential to interpreting the data presented should be made available in the figure legends.<br><br>Have you included all the information requested in your manuscript?	Yes
**Resources**	Yes

A description of all resources used, including antibodies, cell lines, animals and software tools, with enough information to allow them to be uniquely identified, should be included in the Methods section. Authors are strongly encouraged to cite Research Resource Identifiers (RRIDs) for antibodies, model organisms and tools, where possible.<br><br>Have you included the information requested as detailed in our Minimum Standards Reporting Checklist?	
**Availability of data and materials**<br><br>All datasets and code on which the conclusions of the paper rely must be either included in your submission or deposited in publicly available repositories (where available and ethically appropriate), referencing such data using a unique identifier in the references and in the "Availability of Data and Materials" section of your manuscript.<br><br>Have you have met the above requirement as detailed in our Minimum Standards Reporting Checklist?	Yes

Powered by Editorial Manager® and ProduXion Manager® from Aries Systems Corporation

Manuscript

Click here to access/download;Manuscript;GIGA-D-22-00030_R2_NN_AGN_MEGC.pdf

Title

A novel ground truth multispectral image dataset with weight, anthocyanins and brix index measures of grape berries tested for its utility in machine learning pipelines

Authors

Pedro J. Navarro[1], Leanne Miller[1], María Victoria Díaz-Galián[2], Alberto Gila-Navarro[2], Diego J. Aguila[3] , Marcos Egea-Cortines[2]

Affiliations

1. Escuela Técnica Superior de Ingeniería de Telecomunicación (DSIE), Campus Muralla del Mar, s/n, Universidad Politécnica de Cartagena, 30202 Cartagena, Spain.
2. Genética Molecular, Instituto de Biotecnología Vegetal, Edificio I+D+I, Plaza del Hospital s/n, Universidad Politécnica de Cartagena, 30202 Cartagena, Spain.
3. Sociedad Cooperativa Las Cabezuelas, 30840 Alhama de Murcia, Spain.

corresponding author(s): Pedro J. Navarro (pedroj.navarro@upct.es)

Abstract

Background

The combination of computer vision devices such as multispectral cameras coupled with Artificial Intelligence has provided a major leap forward in image-based analysis of biological processes. Supervised Artificial Intelligence algorithms require large ground truth image datasets for model training, which allows to validate or refute research hypotheses and to carryout comparisons between models. However, public datasets of images are scarce and ground truth images are surprisingly few considering the numbers required for training algorithms.

Results

We created a dataset of 1283 multidimensional arrays, using berries from five different grape varieties. Each array has 37 images of wavelengths between 488.38nm and 952.76nm obtained from single berries. Coupled to each multispectral image we added a dataset with measurements including, weight, anthocyanin content and Brix index for each independent grape. Thus, the images have paired measures creating a ground truth dataset. We tested the dataset with two neural network algorithms: multilayer perceptron (MLP), three-dimensional

convolutional neural network (3D-CNN). A perfect (100% accuracy) classification model was fit with either the MLP or 3D-CNN algorithms.

Conclusions

This is the first public dataset of grape ground truth multispectral images. Associated with each multispectral image there are measures of the weight, anthocyanins, and Brix index. The dataset should be useful to develop deep learning algorithms for classification, dimensionality reduction, regression, and prediction analysis.

Context

Traditionally, hyper o multispectral images (MSI) have been acquired from satellites or aircraft for the tasks of classification and detection of ground elements [1], vegetation quantification and evolution [2], measurement of ice at the poles [3], or for the detection and monitoring of man-made discharges [4]. The evolution of hyperspectral capture devices based on the decomposition of light in systems with filters on the imaging sensors has introduced notable improvements in spectral sensing. These include a drastically reduced size of the device. The complex calibration process associated with image capturing using linear devices has been eliminated. The number of images per second has been increased. Finally, it is possible to capture up to 25 bands in different spectral ranges in a single shot. These new features allow spectral imaging to expand to new areas of use that were unthinkable a few years ago, such as: disease [5] or water stress detection [6] in crops from on-board drones or autonomous robots, food inspection [7], material classification [8], cancer diagnosis [9], or plant phenotyping [10], among others. A difference between hyperspectral and multispectral sensing technology is the extent of the reflectance spectrum captured. In hyperspectral sensing a contiguous and continuous spectrum is acquired while in multispectral sensing, only specifically targeted reflectance wavelengths are. In this work we use the latter technology.

Advances in techniques based on Deep Learning (DL), inherited from Artificial Neural Networks (ANNs) which mimic the neural behaviour of the brain, have managed to outperform humans in automatic pattern recognition systems [11]. Among the different DL techniques, special mention should be given to the Convolutional Neural Networks (CNNs) for their flexibility and scalability when solving problems in the field of computer vision. These networks have obtained excellent results in the detection, classification, and segmentation of images. Furthermore, CNNs can be used to solve regression problems simply by modifying the activation functions of the last layers [12].

Supervised learning algorithms must be trained with ground truth images, i.e., images that have been associated with a qualitative or quantitative measurement, also called labels.

Obtaining predictive models using multi or hyperspectral images usually involves two stages. First, the images are converted to feature vectors. This process typically involves the use of image semantic segmentation and feature selection or extraction algorithms. The image segmentation can be carried out by filtering the pixels with a reflectance threshold value for one of the channels. This produces a binary image where object and background pixels are identified [13– 15] . The process can be done manually using image analysis software such as ENVI [16]. Some feature selection algorithms that are used include competitive adaptive reweighted sampling (CARS) [13,15,17,18], the successive projection algorithm (SPA) [13,15,17], the genetic algorithm (GA) [17] or random frog [14]. The well-known principal component analysis (PCA) and deep neural networks like autoencoders and convolutional neural networks (CNN) can also be used for feature extraction [13]. It is also possible to simply calculate the mean reflectance of object pixels for every channel in the image and use it as features.

Supervised learning algorithms are then used to fit predictive models, such as partial least square regression (PLSR), least squares-support vector machines (LS-SVM) or multilayer perceptron (MLP). Some application examples of these algorithms include the prediction of anthocyanin levels in goji berries (*Lycium ruthenicum*) [13], the pectin content of mulberry [19], the sugar content of wine grape berries [20] and Dangshan pears (*Pyrus* sp.) [17], the pigment levels of spinach leaves [14], the soluble solids content of apple peels [18] and the water and capsaicinoid content of chilli peppers [15].

Examples of classification problems related to fruits solved with machine learning algorithms and MSI as input data include the evaluation of injuries in mangoes, with LS-SVM combined with PCA extracted features [21]; the discrimination between naturally and artificially ripened bananas using SVM and Probabilistic Collaborative Representation Classifier (ProCRC) [22]; the detection and classification of citrus green mould using Linear Discriminant Analysis (LDA) [23] or the discrimination of olives fruits based on their firmness with a MLP [24].

As mentioned above, some algorithms can be used to fit both classification and regression models, such as SVM or MLP. The CNN family of algorithms deserves special attention because they are capable of simultaneously extracting features and fitting a classification or regression model. Thus, when such algorithms are used, there is no need to segment or manually convert the input MSI to feature vectors.

Despite the current importance of image analysis, there are very few ground truth datasets publicly available containing multispectral images. A well-documented dataset corresponding to Arabidopsis rosette [25] is found in www.plant-phenotyping.org. However, ground truth

datasets where the concentration of a chemical or metabolite is coupled to images are nonaccessible to the best of our knowledge. The multispectral dataset presented in this manuscript is the first which includes multispectral images in the visible and infrared spectrum combined with weight, anthocyanins, and brix index measurements. The dataset has been designed to be easily used in multispectral image classification with DL methods, dimensionality reduction algorithms based on multispectral images.

Methods

Dataset creation

We collected 150 bunches of five seedless table grape varieties, *AutumRoyal*, *Crimson*, *Itum4*, *Itum5* and *Itum9* to create the dataset. All grapes were collected from the same vineyard, located in the municipality of Alhama de Murcia, in the province of Murcia, in South-East Spain. Grapes were harvested when fully ripe for marketing and export, and samples from the field were used for the study. This was roughly 3-4 weeks after veraison.

Samples were taken from each bunch in three characteristic areas of the bunch, categorized as A, – top, B – middle and C-bottom. The grape berries of every class follow a uniform distribution regarding the area of the bunches they were taken from. Different bunches were used during the sampling to account for the possibility of inter-bunch variance. We took a total of 1283 samples: 199 of *AutumRoyal*, 401 of *Crimson*, 84 of *Itum4*, 504 of *Itum5* and 95 of *Itum9*. Grape berries were cleaned, measured, weighed, and labelled before being introduced into the multispectral chamber to obtain the images. Finally, the labelled berries were sent to the laboratory for anthocyanins and brix index measurements. The workflow for the creation of the dataset is shown in Fig.1

Multispectral images acquisition

The multispectral acquisition process was carried out in a multispectral chamber specifically developed for this purpose, which is illustrated in Fig.2. The chamber is composed of:

1) A configurable aluminium structure of size 1000 × 1000 × 500 mm^3. The design of the structure allows easy positioning and placement of different elements, such as reflective panels, cameras, and the illumination system to prepare different types of experiments.
2) A multispectral illumination system. The illumination has been designed with a cluster of LEDs to cover a broad spectrum of wavelengths from 450nm to 970nm (see Fig. 2d). The power supply to the illumination system is controlled by an analogue electronic controller using a software application.

3) A multispectral acquisition system. The multispectral acquisition system consists of two different snapshot mosaic multispectral cameras from the manufacturer

Photonfocus (Switzerland). The first one (model: MV1-D2048x1088-HS03-96-G2) was used for the acquisition of 12 bands in the visible range of 488 nm–625 nm and the second camera (model: MV1-D2048x1088-HS02-96-G2) performed the acquisition of 25 bands in the R-IR (red-infrared) range of 676 nm–952 nm (see Fig. 2b).

4) A software application system. This has been developed using the LabVIEW programming language. It controls the power supplied to the illumination and multispectral acquisition systems (see Fig. 2c and 2d).

The combined multispectral system acquires 37 raw images (1 byte per pixel) for each grape sample in the spectral range of 430 - 953 nm. We used all the channels despite increased noise in the reflectance of the two end wavelengths, to gather the largest amount of information. The grape samples were captured next to a reference mark with a size of 1 cm^2 for easy transformation of pixel values to real measurements (see Fig. 3). As images were not taken from a zenith perspective, the square appeared as a rhomboid in the images, thus aiding in the automatic measurement. The multispectral raw image (I_r) is calibrated using two reference images captured in two different exposure times ($t_{1,2}$) according to equation 1. The dark reference image (I_d) is obtained using a black surface placed at the position of the object to be acquired. The white reference image (I_w) is obtained using a white surface placed at the same position and with the same configuration of the illumination system used for the acquisition.

$$I_c = \frac{I_r(t_1) - I_d(t_1)}{I_w(t_2) - I_d(t_2)} \qquad (1)$$

The exposure times (t_1, t_2) were selected to avoid saturated pixels in the calibrated image (I_c). Particularly for this dataset, the exposures time was set empirically as $t_1 = 15\ ms$ and $t_2 = 10\ ms$.

Image processing

An example of how the raw MSIs look like is presented in Fig.3. The noticeable lack of uniformity in the intensity of the images due to the reflectance mainly of the black and red berries was found to be problematic. To segment the grapes and remove unwanted objects from the images, an algorithm based on computer vision techniques has been developed to automatically obtain the segmented image of each grape.

The algorithm consists of the following steps:

1. Extraction of binary patterns from the different types of grapes. This was done selecting 100 images of each variety easy to segment and with a high contrast.

2. Scaling of the binary pattern. From each binary pattern a set of variations scaling between 0.2 to 20 with step 1.0 are generated and this is applied to the edge image with the Canny function: $\{EP_0,\ldots,EP_t\}$.

3. MSI preprocessing. To obtain a uniform edge image close to the scaled pattern $\{EP_0,\ldots,EP_t\}$, the following image processing pipeline were applied over every channel of each MSI:

a. Look-at-table with alpha = 2.5
b. Gaussian Filter with window 11x11
c. Adaptative thresholding based on mean with: Size×blockSize=(17.2)
d. Morphological CLOSE function with window 3x3
e. Erase areas less than 500 pixels to avoid small objects
f. Canny function edge detector.

The result is a set of smooth edges images $\{SE_0,\ldots,SE_n\}$ with occlusions per each multispectral image.

4. Matching. A matching function is used to find the grape in the MSI. This is done using multiple instances of the matching function (cv2.matchTemplate(SE_i, EP_j, cv2.TM_CCOEFF)) that are invoked between the set of binary patterns $\{EP_0,\ldots,EP_t\}$ and the smooth edge images set per each multispectral $\{SE_0,\ldots,SE_n\}$. The matching function supplies a coefficient (TM_CCOEFF) for the correlation between each pair of images $\{SE_i, EP_j\}$.

5. Region Of Interest. The maximum value of the TM_CCOEFF will determine the area where the grape is found. The ROI [y:y+h,x:x+w], will be cropped of the all bands of a specific multispectral image; being (y, x) the upper left corner of the rectangle with greater TM_CCOEFF, h is the pattern height, and w the pattern width.

An example of a segmented multispectral image obtained with this algorithm is shown in Fig. 4 (only one reflectance channel shown).

6. Save segmented images and go to step 1

This algorithm had to be developed because a simple threshold segmentation with the raw images as input would not work with the darker berries, namely those of *AutumRoyal* class.

Anthocyanins and brix index measures

Anthocyanins were quantified as described previously [26]. Total anthocyanins were extracted with 5 millilitres of an acidic methanol (0.1%) solution, CH3OH:HCl:H20 (70:0.1:29.9 v/v/v). Samples were incubated at 4ºC in darkness for 24 hours. Then, anthocyanins were measured using two different absorbances (530 and 657 nm) with a spectrophotometer (ion UV 1600, US). The amount of anthocyanin was obtained by using the equation 2:

$$Q_{total\ \mathrm{anthocyanin}} = (A_{530} - 0.25 \times A_{657}) \ / \ FW \tag{2}$$

where A_{530} and A_{657} are the absorbances obtained with the spectrophotometer with a wavelength of 530 nm and 657 nm, respectively. FW is the fresh weight of the sample.
We measured brix index with a digital refractometer (ATAGO PAL-1, Japan) using the grape juice extracted from each berry [27].

Data validation and quality control

Dataset structure

The data, which is stored in *gigadb.org*, consists of a total of 1283 multidimensional arrays in TIF format, compressed in a single zip file. There are 5 different grape varieties in the dataset. The images of *AutumRoyal, Crimson,* and *Itum5* classes have been obtained in several batches on different days. In the case of *Crimson* and *Itum5*, images were acquired in different months. This is important because grape attributes are not uniform all over the year and in fact vary depending on the month. Table 1 shows the composition of the dataset, i.e., the number of instances of each grape class as well over how many days the images were obtained and in which month(s). The *AutumRoyal* is a black grape, *Crimson* is green to lightly red-colored, *Itum4* and *Itum5* are green, while *Itum9* is dark red. The size of this dataset is comparable to the one used in [28] were they use 1260 MSI of grapes of 2 varieties to adjust a classifier capable of predicting the ripeness of grape berries.

Each one of the arrays in the dataset correspond to a MSI, with the following dimensions, in pixel: 140 (height) x 200 (width) x 37 (number of channels/depth). The first 12 channels correspond to reflectance in the visible range (VIS) of the electromagnetic spectrum, and the last 25 ones to near infrared (NIR) reflectance. The specific wavelength of every channel is shown in Table 2

In addition to the images, a ground truth for specific variables in the format of a tab separated text file (.txt) is stored in the repository. For every grape in the dataset, the value of the

following variables is present in this file: the brix index, weight expressed in grams; the amount of anthocyanins expressed in milligrams per kilo of fresh weight; the type of grape and finally an identification of the measured batch. As an overview of this file, the first five rows are shown in Table 3.

Dataset visualization

The first analysis was a dimensionality reduction with the PCA algorithm to gain insight of the internal structure of the dataset. To do that, the dataset was compressed from a collection of multispectral images to a two-dimensional table. This was done by threshold segmentation of the multispectral images and then calculating the mean reflectance across all object pixels for every channel and image. The resulting table has as many rows as there are images in the dataset, and as many columns as reflectance channels. For the image segmentation, the channel 22 (802.25 nm) and the threshold value 25 were used. These parameters were both empirically selected. Once the dataset had been compressed, the PCA algorithm was applied to it.

The first two principal components (PCs) explain up to 84% percent of the total variance of the dataset. More specifically, the first PC was mostly a product of the NIR reflectance channels, and the second PC was formed by the VIS channels. The first PC itself accounts for 54% of the total variance, and, as it is shown in Fig. 5 and can separate most of the instances. This suggests that most of the reflectance of the grape berries is formed by NIR radiation and thus it is within this range of the spectrum where the different varieties can be best discriminated.

Figure 5 illustrates substantial intragroup variance for *AutumRoyal, Crimson* and *Itum5* grape types, while it is less pronounced in *Itum9* and *Itum4*. As mentioned earlier the most likely explanation for this phenomenon is that the images of some classes were captured over more than one day and in different months. This is particularly remarkable for *Itum5* and *Crimson* because they were measured over 3 and 4 months respectively. By contrast, *Itum9* and *Itum4* only were measured in one month and they cluster much closer together.

We also assessed the differences between every grape class via their reflectance spectrogram comparison (Fig.6). In accordance with Fig.5, most of the reflectance was found to be in the NIR range and this was also the region with the most intergroup variance. We selected grapes randomly using the Python library Numpy random sampling method. By plotting the spectrogram of each class, we could also see the heterogeneity of *AutumRoyal*, *Crimson* and *Itum5* (Fig.6)

The spectra obtained from grape berries differ significantly from the better-known leaf spectra. This is due mostly to the differences in chlorophyll content of the tissues. Indeed, the reported concentration of chlorophyll in grape berries at harvest is 1000 fold lower than leaves of spinach, lettuce or pakchoi [29] [30].

Finally, the distribution of the continuous variables present in the ground truth table file was analysed, namely anthocyanin content in mg/kg fresh weight FW and brix index degrees (Fig.7). According to the Shapiro-Wik tests, anthocyanins were not normally distributed in the varieties analysed. *Itum5*, *Itum4* and *Crimson* had leptokurtic distributions while *AutumRoyal* and *Itum9* had platykurtic distributions. The anthocyanin ranges also differed between each grape class. Both *Itum4* and *Itum5* had a very short range, centered around zero [0;0.98] and [0.0073;2.96] respectively while it was much broader in the cases of *AutumRoyal* with [0.63;95.23] and *Itum9* [2.78;95.60]. Finally, *Crimson* ranged between 1.45e-4 and 2.45 mg/kgr fw. These differences were due to the different pigmentation levels of the grapes. *Itum4* and *Itum5* are both green grapes, and thus lack anthocyanin pigments in the skin of the berries. In contrast, *Crimson* is lightly red-colored and therefore possess some pigmentation while *AutumRoyal* and *Itum9* are black or dark red and have the highest level of anthocyanins in their skin.

The brix index distributions were less skewed than anthocyanins (Fig.7). *Crimson* and *Itum4* were normally distributed, according to the result of Shapiro-Wilk tests. Their ranges were also more similar than was the case with the anthocyanin content: [12.8-22] for *Itum4*, [12.6-30.8] for *Itum5*, [15.9-26.6] for *Itum9*, [11.7-26] and *AutumRoyal* and [13.1-25.3]. Interestingly, *AutumRoyal* and to a lesser extent *Itum9* showed a bimodal distribution. As these grapes were measured over a single month each, this may indicate differing levels of ripening in the trusses analyzed (Fig.7).

Potential usage of dataset

Dataset utility in machine learning pipelines

To assess the usefulness of this ground truth data set in machine learning pipelines, we tried unsupervised and supervised pipelines, fitting classification models. We used K-means clustering algorithm for the unsupervised pipeline and multilayer perceptrons (MLP) and convolutional neural networks with 3D kernels (3D-CNN) for the classification models. The goal of the classifiers is to predict the class of the grapes.

Two neural network algorithms were used to fit classification models: MLP and 3D-CNN. MLP requires the data in 2D table form, the same one that was used as input for the PCA algorithm.

The second one can use the 3D arrays of the dataset directly as input, but requires the usage of Data Augmentation techniques, such as those provided in the Python 3 library Albumentations [31].

The MLP used consisted of a simple stacking of 4 dense layers with 32, 24, 16 and 8 nodes each, plus a final output layer with 5 nodes. Between each dense layer, a batch normalization layer and the ReLU activation function were inserted. After the output dense layer, we used the softmax activation function. This network is quick to train, but the input dataset must be preprocessed beforehand, via spatial and spectral compression.

The 3D-CNN architecture employs two 3D-convolutional blocks with convolutional layers that use dilated 3D kernels [32] 5It also uses mean and average 3D pooling layers between these blocks and a total of 2 dense layers. This network requires not only a great number of images to be trained (hence the need of Data Augmentation techniques), it is also slow. It needs to store in memory tensors of considerable size, which is computationally heavy. The network architecture is summarized in Fig.9.

To train both networks, the data was split between three separate subsets called train, validation and test, each one having respectively the 50%, 25% and 25% of the total instances. The split was not done randomly, instead, the class distribution was preserved in all subsets, so that in each one, there was the same proportion of grapes. The train subset is used to fit the model, the validation is used for testing it while it is being fit and the test one is used after the fit has been completed to get a final performance value. By splitting the data like this, the leakage of information between subsets is avoided. In the case of the model fitted with the 3D-CNN algorithm, Data Augmentation was applied to both the train and validation subsets, but not to the test subset. The Data Augmentation consisted in affine transformations, vertical and horizontal flippings and lightly altering the pixel values via contrast and brightness changes. The transformations applied were carefully selected to avoid distorting the reflectance information contained in the images. The flipping of images and affine transformation do not change the pixel reflectance values contained in the pixels. The contrast and brightness do change the pixel values, but its range was limited to minimize any possible hinderance in the learning process of the classifiers. The precise values of the transformation ranges are identical to those described in the literature [32]

In both cases, a perfect (100%) classification accuracy was reached, which validates the quality of this dataset to fit classification models with complex and simple network architectures. The success of these algorithms is likely due to the fact that they are able to find and exploit non linear relationships between the independent variables, i.e. the reflectance spectra.

Indeed, we have also fit a classifier using a simpler algorithm, namely SVM with linear kernel, and we obtained a modest 0.679 accuracy as the best result. This indicates the need for higher complexity learning algorithms to fit models capable of generalizing with this data.

The visible and infrared arrays of the MSI were not identical in regard to the spatial positions of the object pixels, as they were captured with two separate cameras. This was not an obstacle for the fitting of classification models.

The result of the K-means clustering (with K=8 empirically selected) showed that grapes were not cleanly separated. Interestingly, the month in which the images were acquired does indeed introduce significant differences between grapes of the same type. This was evidenced by the fact that the instances of most classes were distributed across different clusters, and that several clusters contain instances of different classes. In particular, *AutumRoyal*, was split into 2 different clusters. *Crimson* was distributed across mainly 4 clusters but only one of these clusters is composed entirely of *Crimson* instances. *Itum4* instances are all located in one cluster, together with *AutumRoyal* instances. *Itum5*, like *Crimson* is split across 4 clusters, but only one of them is comprised entirely of instances of this class. Finally, for *Itum9* only one cluster contained all the instances, together with *Itum5*. Except for *AutumRoyal*, the number of clusters that contain all instances of a given grape class coincided with the number of months over which the images of that class were acquired. The results were visualized over the same PCA scatter plot already shown in Fig. 5 and are presented in Fig.8. These clustering results indicate that a supervised learning algorithm may find problems fitting a classification algorithm, because instances labelled equally can have quite different reflectance spectra depending on the month when the image was captured. In contrast, neural networks achieve 100% accuracy.

An unexpected result of our analysis was the capacity to identify differences between berries based on time of harvest, or position in the truss. This explains the significant intragroup variance in some grape classes. Those were not obstacle for the neural networks used as classification algorithms. Furthermore, our dataset reflects the reality encountered when using

agricultural products, i.e. a large variance due to a combination of environmental, ontogenic and genetic factors.

In addition to classification and clustering, we have also tried to fit regression models capable of predicting either the anthocyanin content or the Brix Index. However, we were unsuccessful in our attempts. We believe that the structure of the data prevents the algorithms to extract meaningful relations between the reflectance and the values of the continuous variables presented (anthocyanin content and Brix Index). The distributions of Anthocyanin content are too different between grape classes. More than three quarters of all grapes measured had little to undetectable anthocyanin levels (*Itum5*, *Itum4* and *Crimson*), while the remaining classes had very high levels (*AutumRoyal* and *Itum9*). Hence the algorithms were challenged to fit a model capable of generalizing. Restricting the problem to only one or a few classes was of no use because the number of instances turned out to be too low for the learning algorithms.

Brix Index posed a different problem to fit regression models. In this case, the distribution of this variable is very similar for every grape class of the dataset. This causes the algorithms to fit a model that systematically predicts the global mean of this variable. They are not capable of linking the information contained in the spectra to the Brix Index.

We have tried two additional algorithms, namely Partial Least Squares Regression (PLSR), Support Vector Machine (SVM) alongside the neural networks presented in the paper adapted for regression problems, and none of them were able to successfully fit a regression model. The highest determination coefficient (R2) was 0.53 for Anthocyanin and 0.24 for Brix Index (Data not shown).

We have obtained 1238 multispectral images from grape berries, comprising 37 channels, thus creating a multidimensional array of 45806 images. Coupled to each grape there are additional data, such as weight, anthocyanin content and brix index. To the best of our knowledge, this is the first data set of ground truth multispectral images of fruits to be made publicly available. We propose it as a benchmark for the plant phenotyping community that uses multispectral images to test different classification algorithms on it.

Availabiliy of Source Code and Requirements

Project name: 3DeepM
Project home page https://github.com/AlbertoGilaNavarro/3DeepM [32]
Operating system: Platform independent
Programming languages: Python

Other requirements: Python version 3.6.8 or higher, Tensorflow version 2.4.1 or higher, Numpy version 1.19.5 or higher, Pandas version 1.1.5 or higher, Matplotlib version 3.3.4 or higher, Albumentations version 0.5.2 or higher, ComputerVision2 (cv2) version 4.5.1 or higher, Skimage version 0.17.2 or higher, Imutils version 0.5.4 or higher
License: GNU General Public License version 3

In our home page the functions for the segmentation process of grape berries and the architectures of the neural networks used in the technical validation, together with usage example of model training and validation scripts are publicly available

Availability of Supporting Data

All data further supporting this work, including snapshots of our code are openly available in the GigaScience repository, GigaDB [33].

Acknowledgements

This research was funded by BFU 2017-88300-C2-1-R to Marcos Egea-Cortines, BFU 201788300-C2-2-R to Pedro J Navarro, CDTI 5117/17CTA-P to Marcos Egea-Cortines and the

“Research Programme for Groups of Scientific Excellence in the Region of Murcia” of the Seneca Foundation (Agency for Science and Technology in the Region of Murcia—19895/GERM/15).

There is no local legislation concerning the acquisition of images from grape berries.

Author contributions

P.J.N and M.E.C designed the experiments, supervised the research, and obtained funding; L.M, and M.V.D.G obtained data; D.A grew, obtained biological samples, and selected representative samples; P.J.N and A.G.N analysed data. P.J.N, L.M, A.G.N and M.E.C wrote first draft. All authors corrected and approved manuscript.

Competing interests

D.A. is a table grape grower. D.A. had no direct influence on the outcome of the experiments. The rest of the authors declare no conflict of interest.

References

1. Lee WS. Citrus greening disease detection using aerial hyperspectral and multispectral imaging techniques. *Journal of Applied Remote Sensing*. SPIE-Intl Soc Optical Eng; 2012; doi: 10.1117/1.jrs.6.063542.

2. Zeng L, Wardlow BD, Xiang D, Hu S, Li D. A review of vegetation phenological metrics extraction using time-series, multispectral satellite data. *Remote Sensing of Environment*. Elsevier Inc.; 2020; doi: 10.1016/j.rse.2019.111511.

3. Witharana C, Bhuiyan MAE, Liljedahl AK, Kanevskiy M, Epstein HE, Jones BM, et al.. Understanding the synergies of deep learning and data fusion of multispectral and panchromatic high resolution commercial satellite imagery for automated ice-wedge polygon detection.
ISPRS Journal of Photogrammetry and Remote Sensing. Elsevier B.V.; 2020; doi: 10.1016/j.isprsjprs.2020.10.010.

4. Khanna S, Santos MJ, Ustin SL, Shapiro K, Haverkamp PJ, Lay M. Comparing the potential of multispectral and hyperspectral data for monitoring oil spill impact. *Sensors (Switzerland)*. MDPI AG; 2018; doi: 10.3390/s18020558.

5. Franke J, Menz G. Multi-temporal wheat disease detection by multi-spectral remote sensing. *Precision Agriculture*. Springer; 2007; doi: 10.1007/s11119-007-9036-y.

6. Zhang L, Zhang H, Niu Y, Han W. Mapping maizewater stress based on UAV multispectral remote sensing. *Remote Sensing*. MDPI AG; 2019; doi: 10.3390/rs11060605.

7. Zhang B, Liu L, Gu B, Zhou J, Huang J, Tian G. From hyperspectral imaging to multispectral imaging: Portability and stability of HIS-MIS algorithms for common defect detection.
Postharvest Biology and Technology. Elsevier B.V.; 2018; doi: 10.1016/j.postharvbio.2017.11.004.

8. De Giglio M, Dubbini M, Cortesi I, Maraviglia M, Parisi EI, Tucci G. Plastics waste identification in river ecosystems by multispectral proximal sensing: a preliminary methodology study. *Water and Environment Journal*. Blackwell Publishing Ltd; 2021; doi: 10.1111/wej.12652.

9. Rey-Barroso L, Burgos-Fernández FJ, Delpueyo X, Ares M, Royo S, Malvehy J, et al.. Visible and extended near-infrared multispectral imaging for skin cancer diagnosis. *Sensors (Switzerland)*. MDPI AG; 2018; doi: 10.3390/s18051441.

10. Moghimi A, Yang C, Marchetto PM. Ensemble Feature Selection for Plant Phenotyping: A Journey from Hyperspectral to Multispectral Imaging. *IEEE Access*. Institute of Electrical and Electronics Engineers Inc.; 2018; doi: 10.1109/ACCESS.2018.2872801.

11. Sengupta S, Basak S, Saikia P, Paul S, Tsalavoutis V, Atiah F, et al.. A review of deep learning with special emphasis on architectures, applications and recent trends. *KnowledgeBased Systems*. 2020; doi: 10.1016/j.knosys.2020.105596.

12. Navarro PJ, Miller L, Rosique F, Fernández-Isla C, Gila-Navarro A. End-to-End Deep Neural Network Architectures for Speed and Steering Wheel Angle Prediction in Autonomous Driving. *Electronics*. Multidisciplinary Digital Publishing Institute; 2021; doi: 10.3390/electronics10111266.

13. Zhang C, Wu W, Zhou L, Cheng H, Ye X, He Y. Developing deep learning based regression approaches for determination of chemical compositions in dry black goji berries (Lycium ruthenicum Murr.) using near-infrared hyperspectral imaging. *Food Chemistry*. 2020; doi: 10.1016/j.foodchem.2020.126536.

14. Zhang C, Wang Q, Liu F, He Y, Xiao Y. Rapid and non-destructive measurement of spinach pigments content during storage using hyperspectral imaging with chemometrics. *Measurement: Journal of the International Measurement Confederation*. 2017; doi: 10.1016/j.measurement.2016.10.058.

15. Jiang J, Cen H, Zhang C, Lyu X, Weng H, Xu H, et al.. Nondestructive quality assessment of chili peppers using near-infrared hyperspectral imaging combined with multivariate analysis. *Postharvest Biology and Technology*. 2018; doi: 10.1016/j.postharvbio.2018.09.003.

16. National Institutes of Health: ImageJ - Image Processing and Analysis Software. https://www.ittvis.com/envi/ Accessed 2021 Aug 4.

17. Zhang D, Xu L, Liang D, Xu C, Jin X, Weng S. Fast Prediction of Sugar Content in Dangshan Pear (Pyrus spp.) Using Hyperspectral Imagery Data. *Food Analytical Methods*. Springer New York LLC; 2018; doi: 10.1007/s12161-018-1212-3.

18. Tian X, Li J, Wang Q, Fan S, Huang W. A bi-layer model for nondestructive prediction of soluble solids content in apple based on reflectance spectra and peel pigments. *Food Chemistry*. 2018; doi: 10.1016/j.foodchem.2017.07.045.

19. Yang L, Gao H, Meng L, Fu X, Du X, Wu D, et al.. Nondestructive measurement of pectin polysaccharides using hyperspectral imaging in mulberry fruit. *Food Chemistry*. 2021; doi: 10.1016/j.foodchem.2020.127614.

20. Gomes VM, Fernandes AM, Faia A, Melo-Pinto P. Comparison of different approaches for the prediction of sugar content in new vintages of whole Port wine grape berries using hyperspectral imaging. *Computers and Electronics in Agriculture*. 2017; doi: 10.1016/j.compag.2017.06.009.

21. Hashim N, Onwude DI, Osman MS. Evaluation of Chilling Injury in Mangoes Using Multispectral Imaging. *Journal of Food Science*. 2018; doi: 10.1111/1750-3841.14127.

22. Vetrekar N, Ramachandra R, Raja KB, Gad RS. Multi-spectral Imaging To Detect Artificial Ripening Of Banana: A Comprehensive Empirical Study. *2019 IEEE International Conference on Imaging Systems and Techniques (IST)*.

23. Ghanei Ghooshkhaneh N, Golzarian MR, Mamarabadi M. Detection and classification of citrus green mold caused by Penicillium digitatum using multispectral imaging. *Journal of the Science of Food and Agriculture*. 2018; doi: 10.1002/jsfa.8865.

24. Martínez Gila DM, Navarro Soto JP, Satorres Martínez S, Gómez Ortega J, Gámez García J. The Advantage of Multispectral Images in Fruit Quality Control for Extra Virgin Olive Oil Production. *Food Anal Methods*. 2022; doi: 10.1007/s12161-021-02099-w.

25. Minervini M, Fischbach A, Scharr H, Tsaftaris SA. Finely-grained annotated datasets for image-based plant phenotyping. *Pattern Recognition Letters*. 2016; doi: 10.1016/j.patrec.2015.10.013.

26. Li Z, Zhao M, Jin J, Zhao L, Xu Z. Anthocyanins and their biosynthetic genes in three novelcolored Rosa rugosa cultivars and their parents. *Plant Physiology and Biochemistry*. 2018; doi: 10.1016/j.plaphy.2018.06.028.

27. Jayasena V, Cameron I. °brix/Acid ratio as a predictor of consumer acceptability of crimson seedless table grapes. *Journal of Food Quality*. 2008; doi: 10.1111/J.1745-4557.2008.00231.X. 28. Ramos RP, Gomes JS, Prates RM, Simas Filho EF, Teruel BJ, dos Santos Costa D. Noninvasive setup for grape maturation classification using deep learning. *Journal of the Science of Food and Agriculture*. 2021; doi: 10.1002/jsfa.10824.

29. Kamffer Z, Bindon KA, Oberholster A. Optimization of a Method for the Extraction and Quantification of Carotenoids and Chlorophylls during Ripening in Grape Berries (*Vitis vinifera* cv. Merlot). *J Agric Food Chem*. 2010; doi: 10.1021/jf1004308.

30. Xue L, Yang L. Deriving leaf chlorophyll content of green-leafy vegetables from hyperspectral reflectance. *ISPRS Journal of Photogrammetry and Remote Sensing*. 2009; doi: 10.1016/j.isprsjprs.2008.06.002.

31. Buslaev A, Iglovikov VI, Khvedchenya E, Parinov A, Druzhinin M, Kalinin AA. Albumentations: fast and flexible image augmentations. *Information*. Multidisciplinary Digital Publishing Institute; 11:1252020;

32. Navarro PJ, Miller L, Gila-Navarro A, Díaz-Galián MV, Aguila DJ, Egea-Cortines M. 3DeepM: An Ad Hoc Architecture Based on Deep Learning Methods for Multispectral Image Classification. *Remote Sensing*. Multidisciplinary Digital Publishing Institute; 2021; doi: 10.3390/rs13040729.

33. Navarro PJ, Miller L, Díaz-Galián MV, Gila-Navarro A, Aguila DJ, Egea-Cortines M (2022): Supporting data for "A novel ground truth multispectral image dataset with weight,

anthocyanins and Brix index measures of grape berries tested for its utility in machine learning pipelines" GigaScience Database. http://dx.doi.org/10.5524/102220

Figure Legends

Figure1: Schematic workflow of the creation of the dataset. Grape bunches from all varieties were harvested after veraison at different months and send to the laboratory. Individual grapes were selected, cleaned and labeled prior to data acquisition. Then the MSI were captured in the chamber. Afterwards, the raw reflectance data was transformed in ready to use 3D arrays and in parallel, we measured the anthocyanin content and brix index.

Figure 2. Multispectral chamber: (a) Illumination system composed of LEDs of different wavelength; (b) Multispectral cameras for visible and IR acquisition; (c) configuration system panel of software application for the cameras setting and to define the parameters of the experiment (d) LEDs illumination system channels' wavelengths and maximum power in Watts. The 760-970 channel is the NIR channel and it is comprised of LEDs of 760, 800, 820, 840, 880, 910, 940 and 970 nm.

Figure 3. Sample of calibrated images in visible and infrared spectrum. The left column contains the reflectance arrays at 542.17 nm and the right column at 767.48 nm. One random instance of each grape class is shown.

Figure 4. Result of the multispectral image segmentation algorithm for a *Crimson* grape. A final segmented image. B binary pattern extracted.

Figure 5: PCA scatter and variable plot (biplot) of the dataset. The class of the instances is represented by the shape of the points and the month when the image was acquired by the color. The variables are represented as black lines with their names next to them. The reflectance of each channel is presented in Table2

Figure 6: Reflectance spectrograms of every grape class. For each class, the mean spectrogram across all instances is presented as well as the individual spectrogram of 50 randomly selected instances.

Figure 7: Density plots of the distribution of the variables anthocyanin content (A) and Brix index (B) for each individual class

Figure 8: PCA scatter plot with color coding for the clusters calculated with the K-means algorithm. The shape of the points represents the grape class.

Figure 9: Schematic representation of the neural networks employed to fit classification models. The term unit which appears both in the MLP (A) and 3D-CNN (B) refers to the number of neurons of a dense layer. In (B) the following abbreviations are used: KS equals kernel size of a convolutional layer; DR equals dilation rate of the kernels of a convolutional layer and PS equals pool size of a pooling layer.

 Click here to access/download;Table;Table1.pdf

Table1: Structure of the dataset

Grape type	Number of images	Acquisition batches	Month(s)
AutumRoyal	199	2	Nov.
Crimson	401	4	Sept.Oct.Nov.Dec.
Itum4	84	1	Sept.
Itum5	504	5	Oct.Nov.Dec.
Itum9	95	1	Sept.

For each grape variety present in the dataset, the following information is presented: The number of instances, over how many days were they acquired and in which month(s).

Table 2: Multispectra Image wavelengths.

**Channel**	**Wavelength (nm)**	**Channel**	**Wavelength (nm)**	**Channel**	**Wavelength (nm)**
1	488.38	14	689.83	27	866.68
2	488.58	15	714.87	28	876.49
3	503.59	16	729.06	29	885.18
4	516.60	17	741.80	30	894.26
5	530.62	18	755.44	31	908.58
6	542.17	19	767.48	32	916.63
7	567.96	20	781.25	33	925.36
8	579.29	21	792.57	34	931.99
9	592.89	22	802.25	35	938.48
10	602.88	23	823.75	36	946.14
11	616.59	24	835.10	37	952.76
12	625.71	25	845.81		
13	676.25	26	856.52		

Table 3: Overview of the ground truth table file. The first five rows are presented

ArrayName	Brix.Index	Grams	Anthocyanins.mg.Kg.FW	Type	Measure
Crimson_37bands_1.TIF	21.7	0.028	4.070	Crimson	Crimson September
Itum9_37bands_1.TIF	18.5	0.100	33.239	Itum9	Itum9 September
Itum5_37bands_1.TIF	20.7	0.066	0.370	Itum5	Itum5 October
AutumRoyal_37bands_1.TIF	11.7	0.077	14.892	AutumRoyal	AutumRoyal 1 November
Itum9_37bands_2.TIF	18.7	0.0882	37.757	Itum9	Itum9 September

Figure

Click here to access/download;Figure;Fig1.pdf

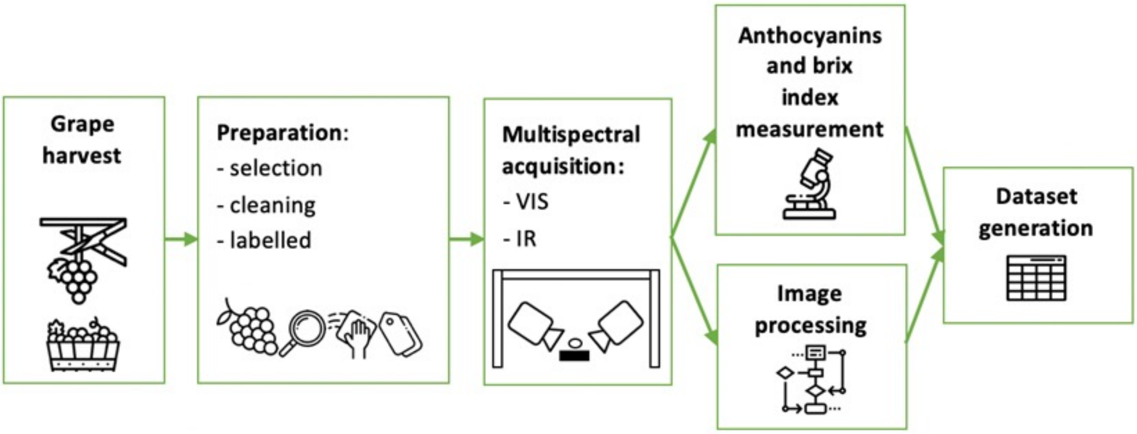

Figure

Click here to access/download;Figure;Fig2Edited2.pdf

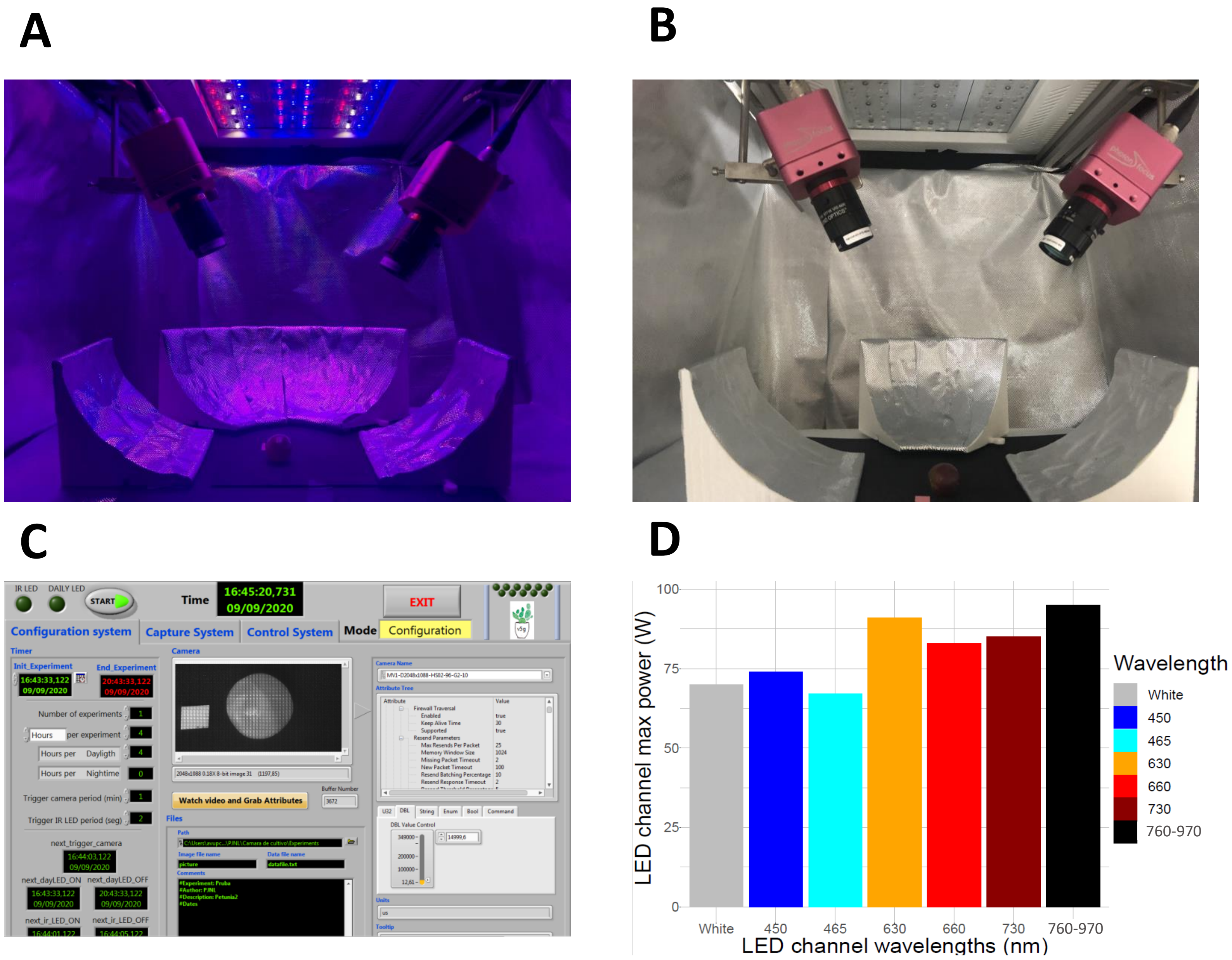

Figure

Click here to access/download;Figure;Fig3.pdf

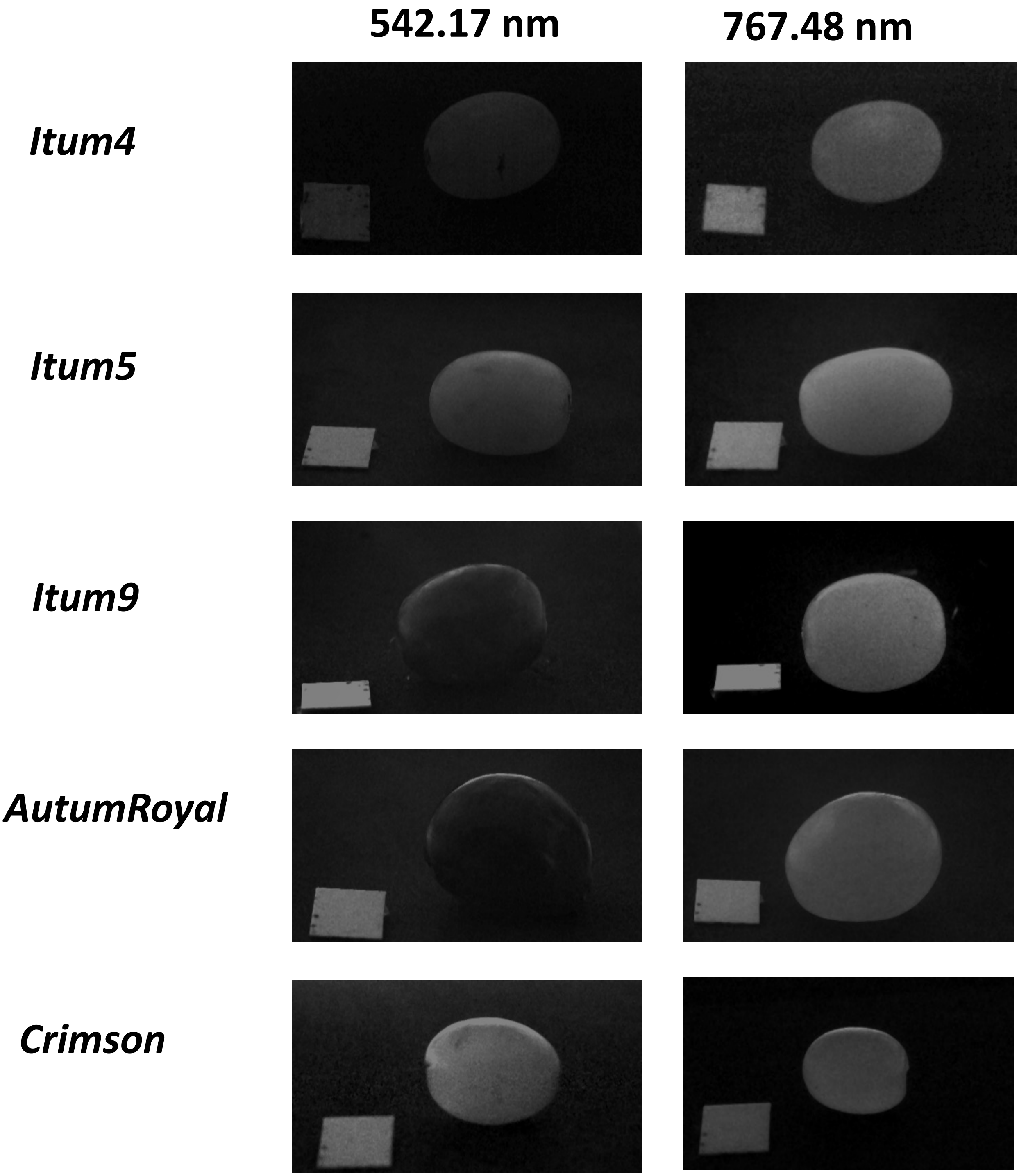

 Click here to access/download;Figure;Fig4.pdf

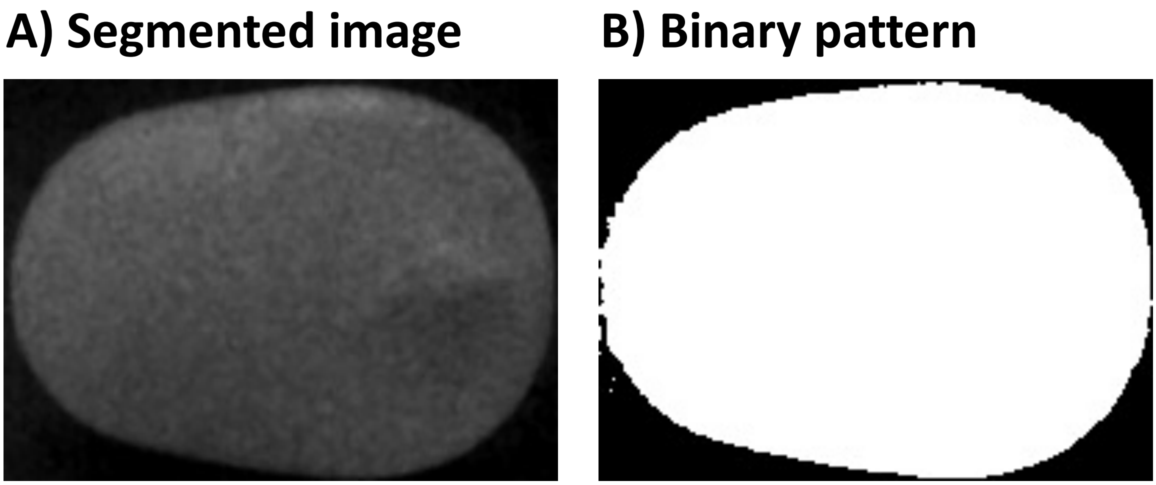

Click here to access/download;Figure;Fig5.pdf

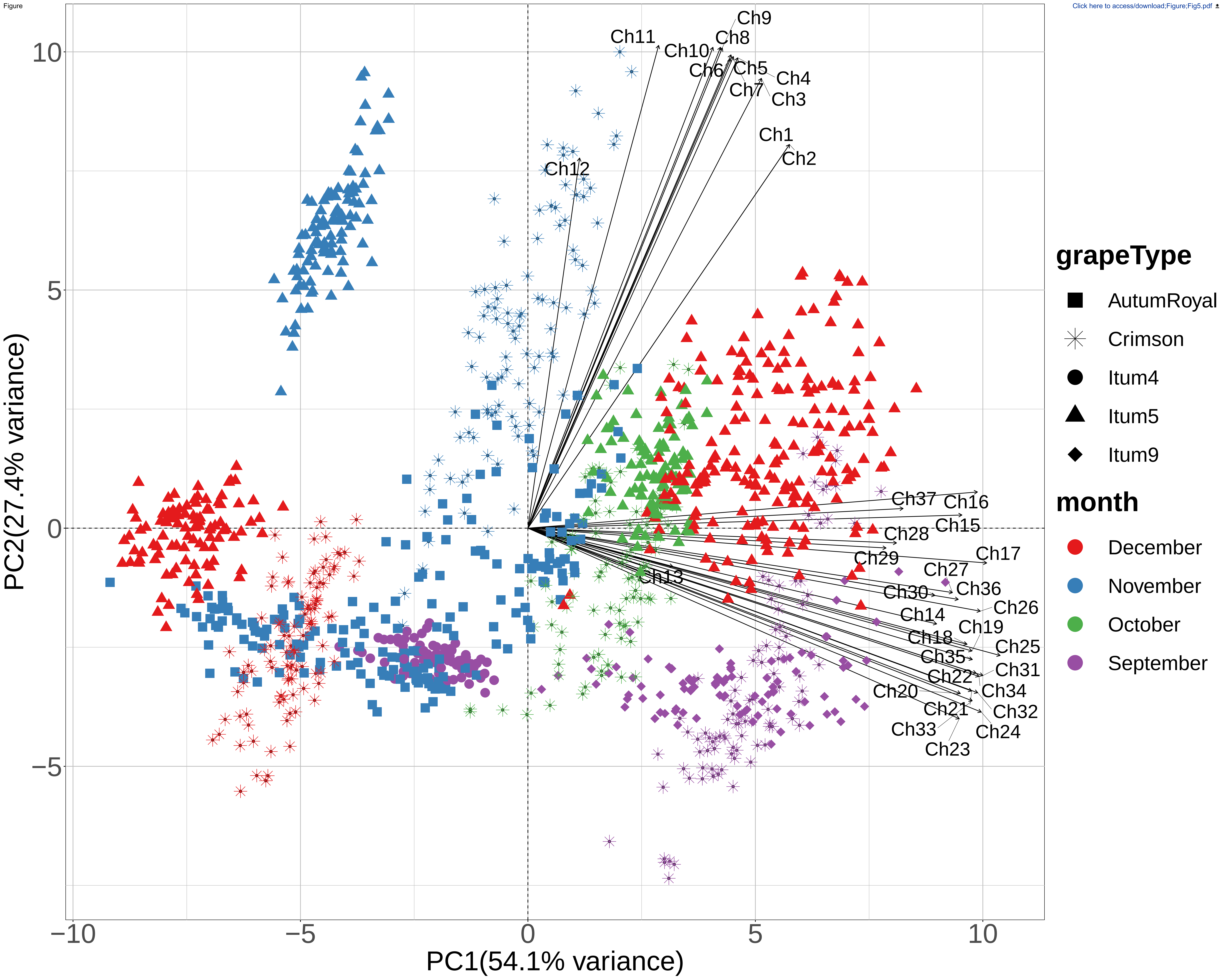

Figure

Click here to access/download;Figure;Fig6.pdf

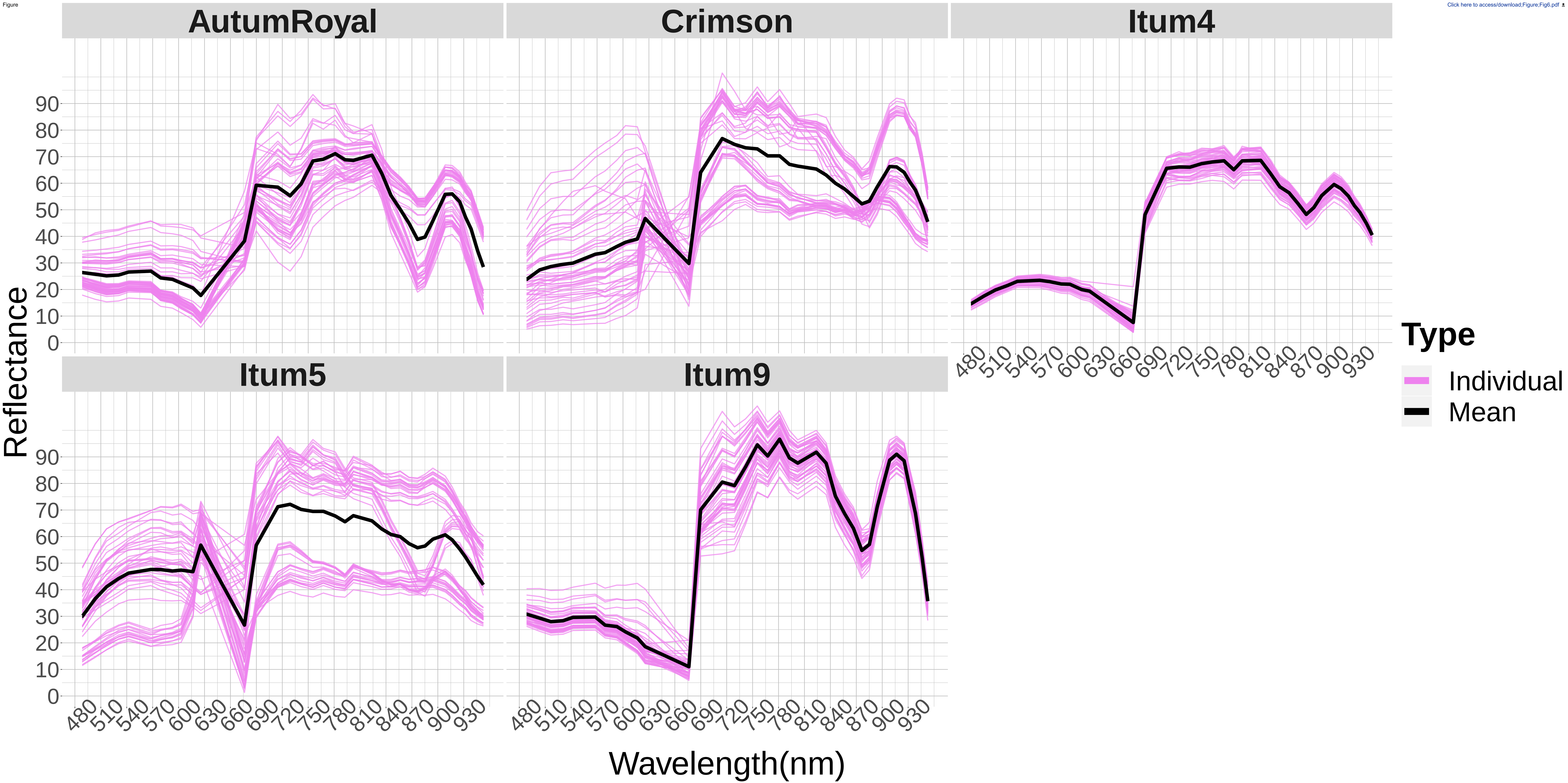

Figure
Click here to access/download;Figure;Fig7.pdf

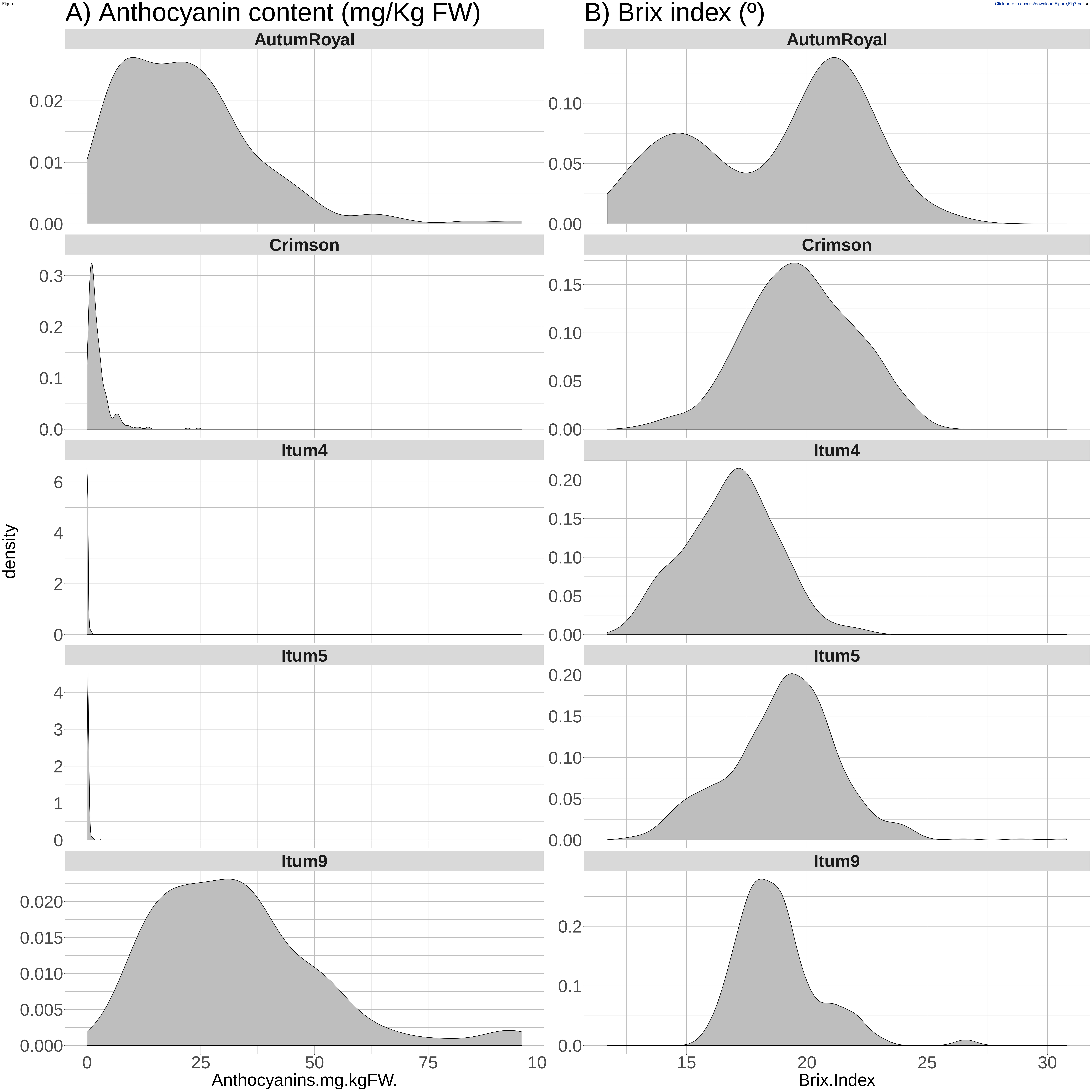

 Click here to access/download;Figure;Fig8.pdf

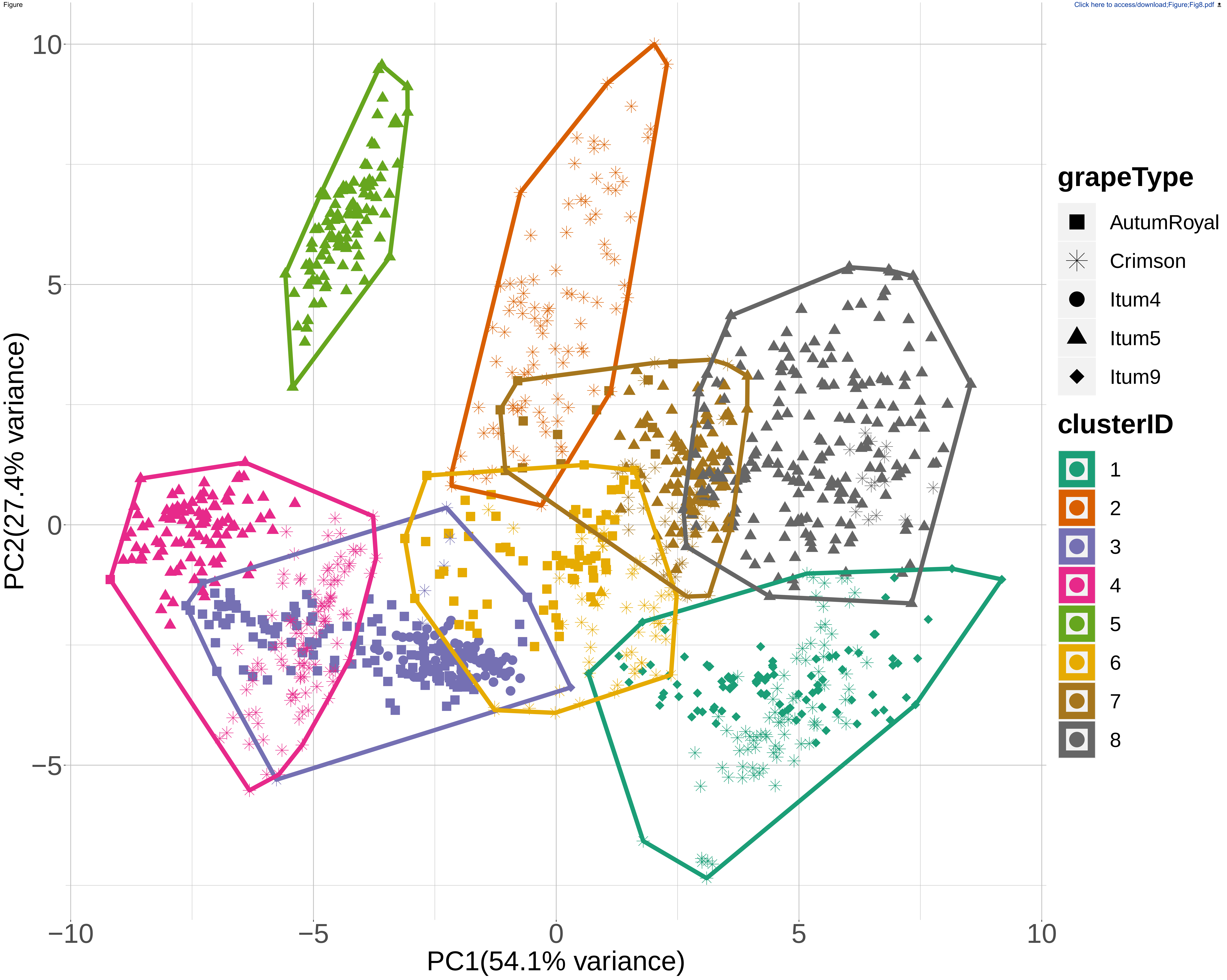

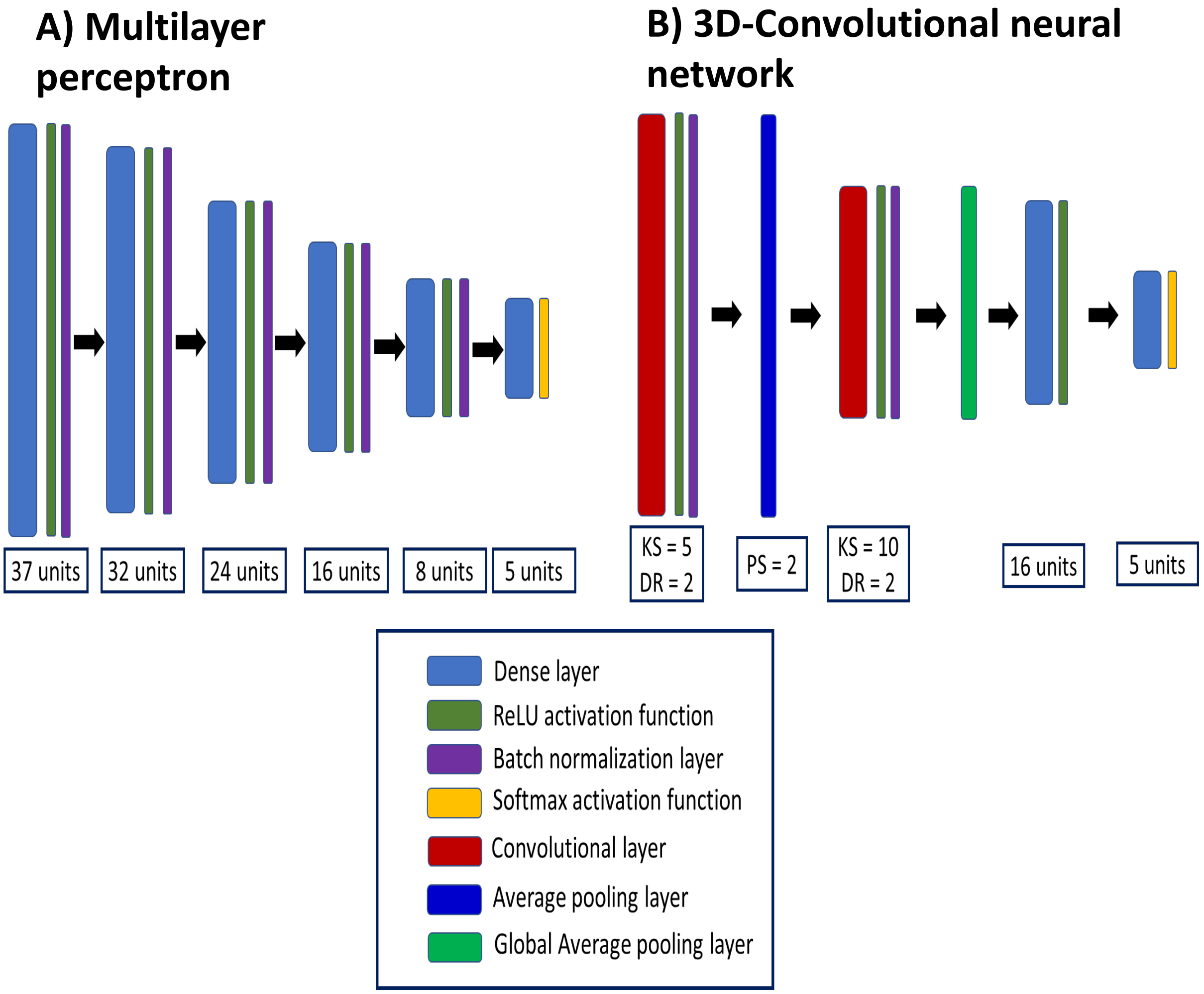
A) Multilayer perceptron
B) 3D-Convolutional neural network
37 units
32 units
24 units
16 units
8 units
5 units
KS = 5
DR = 2
PS = 2
KS = 10
DR = 2
16 units
5 units
Dense layer
ReLU activation function
Batch normalization layer
Softmax activation function
Convolutional layer
Average pooling layer
Global Average pooling layer
