# Supplementary material for: A novel ground truth multispectral image dataset with weight, anthocyanins, and Brix index measures of grape berries tested for its utility in machine learning pipelines
Source: Gigascience. 2022 Jun 14;11:giac052. doi: 10.1093/gigascience/giac052 (PMC9197681; doi:10.1093/gigascience/giac052)
Supplement: giac052_Response_to_Reviewer_Comments_Original_Submission [file giac052_response_to_reviewer_comments_original_submission.pdf]

Author's Response To Reviewer Comments

Close

Dear Dr. Nogoy, first of all thank you very much for the speedy process of refereeing. Second, we appreciate the comments of the three referees. We have modified the manuscript according to their judicious suggestions.

Please find enclosed underneath a point by point response.

I would like to comment that I was supposed to be last author and the system does not let me change position. Is there a way to do it?

Best regards

Marcos

Reviewer reports:

Reviewer #1: This interesting Data Note describes ground truth multispectral images of grape berries, and additionally the 3DeepM software used to train and validate multilayer perceptron (MLP) and three-dimensional convolutional neural network (3D-CNN) classification of these multispectral images. The image data includes 1283 multispectral images of grape berries, and with 37 channels per multispectral image, this creates an impressive multidimensional array of 45,806 images.

In addition, the authors have provided the supporting ground truth data for specific variables referred to in the manuscript, including: brix index, weight (grams), amount of anthocyanins (milligrams per kilo of fresh weight), grape variety, and batch identifier. The authors have additionally provided the tabular data files used to generate the PCA scatter and PCA variable plots shown in the manuscript, and the tabular data file with the average reflectance across every object pixel for every channel and image.

Of note, the software is publicly available from the 3DeepM GitHub repository (https://github.com/AlbertoGilaNavarro/3DeepM) which has been ascribed an OSI-approved GPL-3.0 license.

A minor point is that orthogonal views of the multispectral image data are observed when these data are uploaded in Fiji/ImageJ (i.e. an x-stack rather than a z-stack is the default view of these data in Fiji/ImageJ). This most likely represents a difference between how Matplotlab, as recommended by the authors, and Fiji/ImageJ interprets the axes of these TIFF images.

The correct dimension of the image's arrays are written in lines 244-245, which can serve as a guideline of how any given program must interpret the dimensions.

I recommend this Data Note for publication in GigaScience.

Thanks for your positive response

Reviewer #2: The paper addresses the problem of training data sets for supervised learning models using multispectral grape images. A dataset was created and some tests were performed using it.

Grape data variability is confirmed, which supports the need for larger training data sets (or "less supervised" DL approaches). There are many grape varieties (with implication on the maturity process), and disparate factors that turn classification and, essentially, prediction models using grape data very challenging.

Your dataset includes only 5 varieties. Don't you think that, taking into consideration the data variability involved, is a bit short?

The number of varieties available during a certain timespan in the market is limited due to differing periods of ripening. To the best of our knowledge this is the first dataset created for table grape. It comprises Crimson seedless which is the dominant cultivar in the World with average 30% penetrance, depending on the country. There is a study in grape comprising 2 varieties using multispectral images and the data set is of 1260 images. Thus, the current dataset has a similar size without counting the Data Augmentation transformations of the Albumentation library.

We have added the following to the Data validation and quality control (lines 240-242):

The size of this dataset is comparable to the one used in [1] were they use 1260 MSI of grapes of 2 varieties to adjust a classifier capable of predicting the ripeness of grape berries.

The same applies for the number of samples. Although you've used data augmentation on the multispectral images, there is still a large number of problems depending on the spectra and data augmentation on spectra is not common.

Yes, we consider data augmentation as a tool suitable for any type of image. In fact, the Albumentation algorithm can operate and transform any multidimensional array with n dimensions. Image transformations from Albumentations library can be applied to images of N channels. Rotations, X or Y axis shifting, and scaling do not interfere with the pixel values, i.e, the reflectance information recorded in the images. Changes in brightness do change the pixel values, but we accounted for this and minimize the impact this would pose on the reflectance information by reducing the ranges of this transformation.

We have added the following to Data validation and quality control (lines 351-356):
The transformations applied were carefully selected to avoid distorting the reflectance information contained in the images. The flipping of images and affine transformation do not change the pixel reflectance values contained in the pixels. The contrast and brightness do change the pixel values, but its range was limited to minimize any possible hinderance in the learning process of the classifiers. The precise values of the transformation ranges are identical to those described in the literature [32]
We have added a reference to a previous work were we tested for the first time the 3D-CNN neural network

The characterization of sample choice needs further clarification:
Were the berries collected from the same vineyard? At which location?

We have added the following in Methods (lines 119-120):
All grapes were collected from the same vineyard, located in the municipality of Alhama de Murcia, in the province of Murcia, in South-East Spain.

Were the berries picked from different bunches? Close or distributed in the truss?

In Methods (lines 123-124) we indicated that they were collected from three different areas of the bunches. We also used several bunches per grape variety during sampling. The position of each bunch in the plant used in the generation of the dataset was not recorded as the bunches were harvested for commercial purposes and samples were sent to the laboratory in boxes.

We have added the following to Methods (lines 124-126):

The grape berries of every class follow a uniform distribution regarding the area of the bunches they were taken from. Different bunches were used during the sampling to account for the possibility of inter-bunch variance

:

The time span is detailed in Table 1, but a critical information is missing - veraison.

We have added the following to Methods (lines 121-122) :

Grapes were harvested when fully ripe for marketing and export, and samples from the field were used for the study. This was roughly 3-4 weeks after veraison.
.

Your option to leave all spectral ranges is deliberate (you usually left out the two end wavelenghts - more prone to be noisy)?

We used all ranges, as we consider that using 37 channels is better than 35.

We have added the following to Methods (lines 154-155):
We used all the channels despite increased noise in the reflectance of the two end wavelengths, to gather the largest amount of information.

Did you notice any normalization issues because of dealing with two different acquisition systems?

We did not find normalization issues.

We have added the following to Potential usage of dataset (lines 367-369):

The visible and infrared arrays of the MSI were not identical in regard to the spatial positions of the object pixels, as they were captured with two separate cameras. This was not an obstacle for the fitting of classification models.

Both MLP and 3D-CNN examples presented are classification ones. More challenging ones would be regression problems such as brix or anthocyanin values estimation.

We have added the following paragraphs in Potential usage of dataset:

In addition to classification and clustering, we have also tried to fit regression models capable of predicting either the anthocyanin content or the Brix Index. However, we were unsuccessful in our attempts. We believe that the structure of the data prevents the algorithms to extract meaningful relations between the reflectance and the values of the continuous variables presented (anthocyanin content and Brix Index). The distributions of Anthocyanin content are too different between grape classes. More than three quarters of all grapes measured had little to undetectable anthocyanin levels (Itum5, Itum4 and Crimson), while the remaining classes had very high levels (AutumRoyal and Itum9). Hence the algorithms were challenged to fit a model capable of generalizing. Restricting the problem to only one or a few classes was of no use because the number of instances turned out to be too low for the learning algorithms.

Brix Index posed a different problem to fit regression models. In this case, the distribution of this variable is very similar for every grape class of the dataset. This causes the algorithms to fit a model that systematically predicts the global mean of this variable. They are not capable of linking the information contained in the spectra to the Brix Index.

We have tried two additional algorithms, namely Partial Least Squares Regression (PLSR), Support Vector Machine (SVM) alongside the neural networks presented in the paper adapted for regression problems, and none of them were able to successfully fit a regression model. The highest determination coefficient (R2) was 0.53 for Anthocyanin and 0.24 for Brix Index (Data not shown).

Do you think the classification results will hold with more varieties involved?

We think the dataset can be used to train algorithms and should give excellent results as there is no information transfer between subdatasets.

Reviewer #3: I see the need and potential of your presented dataset. The paper is well written and I appreciate your work.
There are minor recommendations, but in the end I think that you should try to link the spectral data to the measured content information. I would depict a big benefit if you could show this. Even if you can show that it is a hard piece of work it would show that futher scientist can try to solve this.

41: please call it metadata or invasive measured data

Although metadata is used in genomics contexts, the common terminology used in image analysis is ground truth dataset

50: hyperspectral imaging is not always based on filters, please clarify. I recommend to change "multispectral technology" to spectral sensing. Add a second sentence for clarifying the transfer from hyper- to multispectral sensing

We have changed multispectral technology to spectral sensing and added a phrase (lines 58-61):
. A difference between hyperspectral and multispectral sensing technology is the extent of the reflectance spectrum captured. In hyperspectral sensing a contiguous and continuous spectrum is acquired while in multispectral sensing, only specifically targeted reflectance wavelengths are. In this work we use the latter technology.

A difference between hyperspectral and multispectral sensing technology is the extent of the reflectance spectrum captured. In hyperspectral sensing a contiguous and continuous spectrum is acquired while in multispectral sensing, only specifically targeted reflectance wavelengths are. In this work we use the latter technology66: not only regression, but also classification. Why not decision problems?

In line 67 we had stated that CNN algorithms can solve classification, detection, and segmentation problems. All those, together with decision problems, are considered classification problems in the sense that the dependent variable that is modeled is discrete as opposed to regression.

70: you mean labels?

Yes, thank you. We have modified lines 71-72:

Supervised learning algorithms must be trained with ground truth images, i.e. images that have been associated with a qualitative or quantitative measurement, also called labels

73: please clarify: when using CNN whe complete spectral image cube is the input and no segmentation is performed.

We have added the following clarification in Context (lines 101-104):
The CNN family of algorithms deserves special attention because they are capable of simultaneously extracting features and fitting a classification or regression model. Thus, when such algorithms are used, there is no need to segment or manually convert the input MSI to feature vectors.

84-90: this holds for regress but what about classification

We have added the following new examples of classification problems solved with MSI and ML algorithms found in the bibliography (lines 93-101):

Examples of classification problems related to fruits solved with machine learning algorithms and MSI as

input data include the evaluation of injuries in mangoes, with LS-SVM combined with PCA extracted features [21]; the discrimination between naturally and artificially ripened bananas using SVM and Probabilistic Collaborative Representation Classifier (ProCRC) [22]; the detection and classification of citrus green mould using Linear Discriminant Analysis (LDA) [23] or the discrimination of olives fruits based on their firmness with a MLP [24].

As mentioned above, some algorithms can be used to fit both classification and regression models, such as SVM or MLP.

Figure 2: Nice to see your measuring setup. Nevertheless, I will like to see you replace one (C or D) subfigure and add a visualization of the light spectrum of the LED illumination.

Thank you so much for the suggestion

We have replaced subfigure D with a barplot that shows the wavelength of each of the 7 channels of the LED illumination system with their maximum power in Watts. We have also modified figure 2 legend accordingly:

d) LED illumination system channels' wavelengths and maximum power in Watts. The 760-970 channel is the NIR channel, and it is comprised of LEDs of 760, 800, 820, 840, 880, 910, 940 and 970 nm.

143: Please explain why you use two different exposure times, in my opinion it does not make sense. I usually recommend to use the same for imaging and white referencing. Using two different times leads to a reduced/maximed sensor answer.

We agree with the referee, however, we had to optimize the exposure times according to the differing wavelength acquisitions based on a small set of samples, that were discarded due to low quality image.

166: alpha

We have changed it. It is now in line 188

169: please use the right terminus of dilatation and erosion

The right terms are dilation and erosion, and the pipe operation of dilation followed by erosion is called "Closing"

https://docs.opencv.org/3.4/d9/d61/tutorial_py_morphological_ops.html

Figure 5: I see everything, but its hard to read. I recommend to show the scree plot, I feel more comfortable... but it is not a must.

We do not think a scree plot would add extra valuable information, since in Fig.5 the axis already displays the percentage of variance the components 1 and 2 cover. The sum of variance explained by components 1 and 2 is over 80% of the total variance.----- It is written in line 267

197: ml - milliliters

We have changed it. It is in line 219 now

199: please give more information about the instrument, company, country.???

The spectrophotometer and refractometer product names are in lines 221-222 and 227. The multispectral camaras product names are in line 145

We have added the country were each company is based:

Spectrophotometer ion UV 1600 Germany
Refractometer ATAGO PAL-1 Japan
Photonfocus MV1-D2048x1088-HS03-96-G2-10 and MV1-D2048x1088-HS02-96-G2-10 , Switzerland

Fig.6: I wonder why ltum5 does not show a steep ascent in the red edge.... please give information in the discussion part about that. some spectra are not as I expected plant spectra. High after red edge, low before.....

We think that the spectra reflects the fact that grape berries although green, are not truly photosynthetic, and the amount of chlorophyll is three orders of magnitude lower.

We have added the following to Data validation and quality control (lines 288-291)
The spectra obtained from grape berries differ significantly from the better known leaf spectra.
This is due mostly to the differences in chlorophyll content of the tissues. Indeed, the reported concentration of chlorophyll in grape berries at harvest is 1000 fold lower than leaves of spinach, lettuce or pakchoi [2][3]

309: mistake... you mean Fig. 9
Thank you
We have corrected it

322: 100% accuracy is alway tricky. Can you provide a low-complexity model like linear SVM or Random Forest to show that the problem is not easy to solve. If your classification model can be solved by a linear model it is clear that a CNN is performing also well.
Many publications show CNN or other complex networks and show their high accuracy results... But most of these routines are used, because the always work good when there is enough data.

We have fit a classifier using linear SVM with the same feature vectors used as input for the PCA analysis and the MLP (the mean reflectance across every object pixel per every channel, per MSI). The data was split into train and test subsets in the same way the were for the MLP model fitting. We obtained a modest 0.679 of accuracy as a result.

We have added the following in Potential usage of dataset (lines 363-365):

Indeed, we have also fit a classifier using a simpler algorithm, namely SVM with linear kernel, and we obtained a modest 0.679 accuracy as the best result. This indicates the need for higher complexity learning algorithms to fit models capable of generalizing with this data.

286: please clarify what you classify. Classification goal is a assignment to the varieties? So you have a 5 class problem? Is this right? Please clarify!

We have added the following in Potential usage of dataset (line 319-320):
The goal of the classifiers is to predict the class of the grapes.

327: why do you use K = 8 when you just have 5 classes? I think it does make more sense to use K = 5 and to show which clusters are identical with the grape varieties. In this way you can show the similarity between the classes.

We agree with you that K= would be a good value to show intragroup variability in grapes. But the same can be obtained with K=8.

The value of K has been empirically selected to allow for the formation of the most cohesive clusters. The clusters cohesion has not been explicitly calculated with the average silhouette value, but was estimated visually. The point of the unsupervised approach was to show that by looking at the data by itself, the grapes should be classified in more than the 5 classes they belong to. Thus the need of machine learning algorithms that are capable of extracting non linear relationships between the spectral data in order to fit a classifier that can generalize.

472: please add more describing text for figure 1.

We have included the following in Fig.1 :

Grape bunches from all varieties were harvested at different months and sent to the laboratory. There the individual grapes were selected, cleaned and labeled prior to data acquisition. Then the MSI were captured in the chamber. The raw reflectance data was transformed in ready to use 3D arrays and in parallel, the anthocyanin content and brix index was measured

General questions:
Is the calibration data, white referencing and dark current included in the dataset?`
The more information about the calibration is included the more interesting is the publication and the dataset. Please make sure that this data is included.

We have sent to gigaDB all the calibration data

Why is a regression approach with the goal to predict the grape content not shown? I think with just a little more effort it would add a significant plus to the publicated dataset. It seems to be a low hanging fruit.

The regression approach did not work because the reflectance of the different grapes, especially green vs pale red vs red is very different, but brix index is very similar. We believe this confounds the regression algorithms.

We have added the following paragraph in Potential usage of dataset (Lines-396- 416):
We have tried to fit regression models with no success. We believe that the structure of the data prevents the algorithms to extract meaningful relations between the reflectance and the values of the continuous variables presented (Anthocyanin content and Brix Index). The distributions of Anthocyanin content are too different between grape classes. More than three quarters of all grapes measured had little to undetectable Anthocyanin levels (Itum5, Itum4 and Crimson), while the remaining classes had very high levels (AutumRoyal and Itum9). Hence the algorithms were challenged to fit a model capable of generalizing. Restricting the problem to only one or a few classes was of no use because the number of instances turned out to be too low for the learning algorithms.

Brix Index posed a different problem to fit regression models. In this case, the distribution of this variable is very similar for every grape class of the dataset. This causes the algorithms to fit a model that systematically predicts the global mean of this variable. They are not capable of linking the information contained in the spectra to the Brix Index.

We have tried two additional algorithms, namely Partial Least Squares Regression (PLSR), Support Vector Machine (SVM) alongside the neural networks presented in the paper adapted for regression problems, and none of them were able to successfully fit a regression model. The highest R2 was 0.53 for Anthocyanin (Data not shown)

Close
