# Supplementary material for: A novel ground truth multispectral image dataset with weight, anthocyanins, and Brix index measures of grape berries tested for its utility in machine learning pipelines
Source: Gigascience. 2022 Jun 14;11:giac052. doi: 10.1093/gigascience/giac052 (PMC9197681; doi:10.1093/gigascience/giac052)
Supplement: giac052_Response_to_Reviewer_Comments_Revision_1 [file giac052_response_to_reviewer_comments_revision_1.pdf]

Author's Response To Reviewer Comments

Close

Thanks for the review. Concerning the veraison exact dates, there is no such a thing in large orchards. Grapes show a veraison time for a given variety that may take up to a month, depending on the terroir. Thus we think that stating that grapes were taken to the lab when ripe is the best approximation to this issue.

Close
