# Supplementary material for: A novel ground truth multispectral image dataset with weight, anthocyanins, and Brix index measures of grape berries tested for its utility in machine learning pipelines
Source: Gigascience. 2022 Jun 14;11:giac052. doi: 10.1093/gigascience/giac052 (PMC9197681; doi:10.1093/gigascience/giac052)
Supplement: giac052_Reviewer_1_Report_Original_Submission — Chris Armit -- 3/8/2022 Reviewed [file giac052_reviewer_1_report_original_submission.pdf]

**Reviewer Report**

**Title: A novel ground truth multispectral image dataset with weight, anthocyanins and brix index measures of grape berries tested for its utility in machine learning pipelines**

**Version: Original Submission** **Date:** 3/8/2022

**Reviewer name: Chris Armit**

**Reviewer Comments to Author:**

This interesting Data Note describes ground truth multispectral images of grape berries, and additionally the 3DeepM software used to train and validate multilayer perceptron (MLP) and three-dimensional convolutional neural network (3D-CNN) classification of these multispectral images. The image data includes 1283 multispectral images of grape berries, and with 37 channels per multispectral image, this creates an impressive multidimensional array of 45,806 images.
In addition, the authors have provided the supporting ground truth data for specific variables referred to in the manuscript, including: brix index, weight (grams), amount of anthocyanins (milligrams per kilo of fresh weight), grape variety, and batch identifier. The authors have additionally provided the tabular data files used to generate the PCA scatter and PCA variable plots shown in the manuscript, and the tabular data file with the average reflectance across every object pixel for every channel and image.
Of note, the software is publicly available from the 3DeepM GitHub repository (https://github.com/AlbertoGilaNavarro/3DeepM) which has been ascribed an OSI-approved GPL-3.0 license.
A minor point is that orthogonal views of the multispectral image data are observed when these data are uploaded in Fiji/ImageJ (i.e. an x-stack rather than a z-stack is the default view of these data in Fiji/ImageJ). This most likely represents a difference between how Matplotlab, as recommended by the authors, and Fiji/ImageJ interprets the axes of these TIFF images.
I recommend this Data Note for publication in GigaScience.

**Level of Interest**

Please indicate how interesting you found the manuscript: Choose an item.

**Quality of Written English**

Please indicate the quality of language in the manuscript: Choose an item.

**Declaration of Competing Interests**

Please complete a declaration of competing interests, considering the following questions:

- Have you in the past five years received reimbursements, fees, funding, or salary from an organisation that may in any way gain or lose financially from the publication of this manuscript, either now or in the future?
- Do you hold any stocks or shares in an organisation that may in any way gain or lose financially from the publication of this manuscript, either now or in the future?
- Do you hold or are you currently applying for any patents relating to the content of the manuscript?
- Have you received reimbursements, fees, funding, or salary from an organization that holds or has applied for patents relating to the content of the manuscript?
- Do you have any other financial competing interests?
- Do you have any non-financial competing interests in relation to this paper?

If you can answer no to all of the above, write 'I declare that I have no competing interests' below. If your reply is yes to any, please give details below.

I work for GigaScience where I perform checks on the quality of image data and the image analysis approach. My funding is not dependent on the outcome of this review, and I declare that I have no competing interests.

I agree to the open peer review policy of the journal. I understand that my name will be included on my report to the authors and, if the manuscript is accepted for publication, my named report including any attachments I upload will be posted on the website along with the authors' responses. I agree for my report to be made available under an Open Access Creative Commons CC-BY license (http://creativecommons.org/licenses/by/4.0/). I understand that any comments which I do not wish to be included in my named report can be included as confidential comments to the editors, which will not be published.

Choose an item.

To further support our reviewers, we have joined with Publons, where you can gain additional credit to further highlight your hard work (see: https://publons.com/journal/530/gigascience). On publication of this paper, your review will be automatically added to Publons, you can then choose whether or not to claim your Publons credit. I understand this statement.

Yes Choose an item.
