# Supplementary material for: A novel ground truth multispectral image dataset with weight, anthocyanins, and Brix index measures of grape berries tested for its utility in machine learning pipelines
Source: Gigascience. 2022 Jun 14;11:giac052. doi: 10.1093/gigascience/giac052 (PMC9197681; doi:10.1093/gigascience/giac052)
Supplement: giac052_Reviewer_2_Report_Original_Submission — I.G.C.P. Melo-Pinto -- 3/22/2022 Reviewed [file giac052_reviewer_2_report_original_submission.pdf]

Reviewer Report

Title: A novel ground truth multispectral image dataset with weight, anthocyanins and brix index measures of grape berries tested for its utility in machine learning pipelines

Version: Original Submission **Date:** 3/22/2022

Reviewer name: I.G.C.P. Melo-Pinto

Reviewer Comments to Author:

The paper addresses the problem of training data sets for supervised learning models using multispectral grape images. A dataset was created and some tests were performed using it.
Grape data variability is confirmed, which supports the need for larger training data sets (or "less supervised" DL approaches). There are many grape varieties (with implication on the maturity process), and disparate factors that turn classification and, essentially, prediction models using grape data very challenging.
Your dataset includes only 5 varieties. Don't you think that, taking into consideration the data variability involved, is a bit short?
The same applies for the number of samples. Although you've used data augmentation on the multispectral images, there is still a large number of problems depending on the spectra and data augmentation on spectra is not common.
The characterization of sample choice needs further clarification:
Were the berries collected from the same vineyard? At which location?
Were the berries picked from different bunches? Close or distributed in the truss?
The time span is detailed in Table 1, but a critical information is missing - veraison.
Your option to leave all spectral ranges is deliberate (you usually left out the two end wavelenghts - more prone to be noisy)?
Did you notice any normalization issues because of dealing with two different acquisition systems?
Both MLP and 3D-CNN examples presented are classification ones. More challenging ones would be regression problems such as brix or anthocyanin values estimation.
Do you think the classification results will hold with more varieties involved?

Level of Interest

Please indicate how interesting you found the manuscript: Choose an item.

Quality of Written English

Please indicate the quality of language in the manuscript: Choose an item.

Declaration of Competing Interests

Please complete a declaration of competing interests, considering the following questions:

- Have you in the past five years received reimbursements, fees, funding, or salary from an organisation that may in any way gain or lose financially from the publication of this manuscript, either now or in the future?
- Do you hold any stocks or shares in an organisation that may in any way gain or lose financially from the publication of this manuscript, either now or in the future?
- Do you hold or are you currently applying for any patents relating to the content of the manuscript?
- Have you received reimbursements, fees, funding, or salary from an organization that holds or has applied for patents relating to the content of the manuscript?
- Do you have any other financial competing interests?
- Do you have any non-financial competing interests in relation to this paper?

If you can answer no to all of the above, write 'I declare that I have no competing interests' below. If your reply is yes to any, please give details below.

I declare that I have no competing interests

I agree to the open peer review policy of the journal. I understand that my name will be included on my report to the authors and, if the manuscript is accepted for publication, my named report including any attachments I upload will be posted on the website along with the authors' responses. I agree for my report to be made available under an Open Access Creative Commons CC-BY license (http://creativecommons.org/licenses/by/4.0/). I understand that any comments which I do not wish to be included in my named report can be included as confidential comments to the editors, which will not be published.

Choose an item.

To further support our reviewers, we have joined with Publons, where you can gain additional credit to further highlight your hard work (see: https://publons.com/journal/530/gigascience). On publication of this paper, your review will be automatically added to Publons, you can then choose whether or not to claim your Publons credit. I understand this statement.

Yes Choose an item.
