# Supplementary material for: A novel ground truth multispectral image dataset with weight, anthocyanins, and Brix index measures of grape berries tested for its utility in machine learning pipelines
Source: Gigascience. 2022 Jun 14;11:giac052. doi: 10.1093/gigascience/giac052 (PMC9197681; doi:10.1093/gigascience/giac052)
Supplement: giac052_Reviewer_3_Report_Original_Submission — Stefan Paulus, Ph.D. -- 3/24/2022 Reviewed [file giac052_reviewer_3_report_original_submission.pdf]

**Reviewer Report**

**Title: A novel ground truth multispectral image dataset with weight, anthocyanins and brix index measures of grape berries tested for its utility in machine learning pipelines**

**Version: Original Submission** **Date:** 3/24/2022

**Reviewer name: Stefan Paulus, Ph.D.**

**Reviewer Comments to Author:**

I see the need and potential of your presented dataset. The paper is well written and I appreciate your work.
There are minor recommendations, but in the end I think that you should try to link the spectral data to the measured content information. I would depict a big benefit if you could show this. Even if you can show that it is a hard piece of work it would show that futher scientist can try to solve this.
41: please call it metadata or invasive measured data
50: hyperspectral imaging is not always based on filters, please clarify. I recommend to change "multispectral technology" to spectral sensing. Add a second sentence for clarifying the transfer from hyper- to multispectral sensing
66: not only regression, but also classification. Why not decision problems?
70: you mean labels?
73: please clarify: when using CNN whe complete spectral image cube is the input and no segmentation is performed.
84-90: this holds for regress but what about classification
Figure 2: Nice to see your measuring setup. Nevertheless, I will like to see you replace one (C or D) subfigure and add a visualization of the light spectrum of the LED illumination.
143: Please explain why you use two different exposure times, in my opinion it does not make sense. I usually recommend to use the same for imaging and white referencing. Using two different times leads to a reduced/maximed sensor answer.
166: alpha
169: please use the right terminus of dilatation and erosion
Figure 5: I see everything, but its hard to read. I recommend to show the scree plot, I feel more comfortable... but it is not a must.
197: ml - milliliters
199: please give more information about the instrument, company, country.
Fig.6: I wonder why Itum5 does not show a steep ascent in the red edge.... please give information in the discussion part about that. some spectra are not as I expected plant spectra. High after red edge, low before.....
309: mistake... you mean Fig. 9
322: 100% accuracy is alway tricky. Can you provide a low-complexity model like linear SVM or Random Forest to show that the problem is not easy to solve. If your classification model can be solved by a linear model it is clear that a CNN is performing also well.

Many publications show CNN or other complex networks and show their high accuracy results... But most of these routines are used, because the always work good when there is enough data.
286: please clarify what you classify. Classification goal is a assignment to the varieties? So you have a 5 class problem? Is this right? Please clarify!
327: why do you use K = 8 when you just have 5 classes? I think it does make more sense to use K = 5 and to show which clusters are identical with the grape varieties. In this way you can show the similarity between the classes.
472: please add more describing text for figure 1.
General questions:
Is the calibration data, white referencing and dark current included in the dataset?`
The more information about the calibration is included the more interesting is the publication and the dataset. Please make sure that this data is included.
Why is a regression approach with the goal to predict the grape content not shown? I think with just a little more effort it would add a significant plus to the publicated dataset. It seems to be a low hanging fruit.

**Level of Interest**

Please indicate how interesting you found the manuscript: Choose an item.

**Quality of Written English**

Please indicate the quality of language in the manuscript: Choose an item.

**Declaration of Competing Interests**

Please complete a declaration of competing interests, considering the following questions:

- Have you in the past five years received reimbursements, fees, funding, or salary from an organisation that may in any way gain or lose financially from the publication of this manuscript, either now or in the future?
- Do you hold any stocks or shares in an organisation that may in any way gain or lose financially from the publication of this manuscript, either now or in the future?
- Do you hold or are you currently applying for any patents relating to the content of the manuscript?
- Have you received reimbursements, fees, funding, or salary from an organization that holds or has applied for patents relating to the content of the manuscript?
- Do you have any other financial competing interests?
- Do you have any non-financial competing interests in relation to this paper?

If you can answer no to all of the above, write 'I declare that I have no competing interests' below. If your reply is yes to any, please give details below.

'I declare that I have no competing interests'

I agree to the open peer review policy of the journal. I understand that my name will be included on my report to the authors and, if the manuscript is accepted for publication, my named report including any attachments I upload will be posted on the website along with the authors' responses. I agree for my report to be made available under an Open Access Creative Commons CC-BY license (http://creativecommons.org/licenses/by/4.0/). I understand that any comments which I do not wish to be included in my named report can be included as confidential comments to the editors, which will not be published.

Choose an item.

To further support our reviewers, we have joined with Publons, where you can gain additional credit to further highlight your hard work (see: https://publons.com/journal/530/gigascience). On publication of this paper, your review will be automatically added to Publons, you can then choose whether or not to claim your Publons credit. I understand this statement.

Yes Choose an item.
